# Silencing *KIF14* reverses acquired resistance to sorafenib in hepatocellular carcinoma

**DOI:** 10.18632/aging.104028

**Published:** 2020-11-16

**Authors:** Qiankun Zhu, Haiyang Ren, Xiaodong Li, Bo Qian, Shengjie Fan, Fengli Hu, Lishan Xu, Bo Zhai

**Affiliations:** 1 Department of Surgical Oncology and Hepatobiliary Surgery, The Fourth Affiliated Hospital of Harbin Medical University, Harbin 150001, Heilongjiang, China; 2 Department of Gastroenterology, The Fourth Affiliated Hospital of Harbin Medical University, Harbin 150001, Heilongjiang, China

**Keywords:** hepatocellular carcinoma, sorafenib, human kinesin family member 14, transcription factor E26 transformation specific sequence 1, phosphatidylinositol 3-kinase/AKT signalling pathway

## Abstract

For nearly a decade, sorafenib has served as a first-line chemotherapeutic drug for the treatment of hepatocellular carcinoma (HCC), but it displays only limited efficacy against advanced drug-resistant HCC. Regorafenib, the first second-line drug approved for treatment after sorafenib failure, can reverse resistance to sorafenib. We used bioinformatics methods to identify genes whose expression was differentially induced by sorafenib and regorafenib in HCC. We identified *KIF14* as an oncogene involved in the acquired resistance to sorafenib in HCC and investigated its potential as a target for reversing this resistance. Sustained exposure of resistant HCC cells to sorafenib activated the AKT pathway, which in turn upregulated KIF14 expression by increasing expression of the transcription factor ETS1. Silencing *KIF14* reversed the acquired resistance to sorafenib by inhibiting AKT activation and downregulating ETS1 expression by blocking the AKT–ETS1–KIF14 positive feedback loop. Moreover, injection of *siKIF14* with sorafenib suppressed growth of sorafenib-resistant HCC tumors in mice. These results demonstrate that targeting KIF14 could be an effective means of reversing sorafenib failure or strengthening sorafenib’s antitumor effects.

## INTRODUCTION

Liver cancer is the sixth most diagnosed cancer worldwide, and the second most frequent cause of cancer-related deaths in males. Hepatocellular carcinoma (HCC) accounts for 75% to 85% of the total liver cancer burden [1]. Although several surgical treatments, including surgical resection, liver transplantation, and radiofrequency ablation (RFA), can dramatically prolong the median overall survival (OS) of patients with early-stage HCC to 60 months, the limited number of therapeutic options for advanced-stage cancer remains the primary challenge. Studies have reported that 80% of patients with HCC are diagnosed at an advanced stage and therefore cannot be treated using the available therapeutic options [1, 2]. To date, four drugs targeting tyrosine kinases have been approved for the treatment of HCC [3]. Sorafenib is the only Food and Drug Administration (FDA)-approved drug for the systemic treatment of advanced HCC for almost a decade [4, 5]. Recently, lenvatinib [6], as an alternative first-line drug (noninferior to sorafenib), and regorafenib [7] and cabozantinib [8], as second-line drugs in case of sorafenib failure, have been approved for the treatment of HCC. However, these have not been widely accepted due to the lack of superiority over sorafenib. Moreover, sorafenib remains the preferred drug for the treatment of patients with advanced HCC. However, drug resistance to angiogenesis inhibitors, including sorafenib, is common in HCC and greatly minimizes the therapeutic benefits of this drug.

Although few HCC cells are initially resistant to sorafenib due to genetic heterogeneity, more cells develop acquired resistance because long-term exposure to the drug reduces their sensitivity to it [9]. Since the approval of sorafenib, extensive studies have been conducted to explore the drug-resistance mechanism(s); several pathways, such as phosphatidylinositol 3-kinase (PI3K)/AKT and JAK–STAT, hypoxia-inducible, autophagy, epithelial–mesenchymal transition (EMT) pathway, and Keap/Nrf2 pathway, have been implicated [9–12]. In addition to alternative pro-angiogenic growth factors, the angiogenesis-independent mechanism contributes to tumor growth [13]. The acquired resistance to sorafenib can be induced by switching tumor growth from angiogenesis-dependent to angiogenesis-independent manner [14]. Moreover, angiogenesis-independent tumor growth is regulated by cell motility, adhesion, and tumor stroma, and may determine the specific resistance pathway [13]. Unfortunately, limited clinical benefits of drugs targeting single components of the above pathways restrict their use as therapeutics [3, 15]. Multitarget tyrosine kinase inhibitors, such as regorafenib and cabozantinib, are still the main second-line treatment options for HCC if the first-line sorafenib fails [3]. Although regorafenib is structurally similar to sorafenib and its targets are similar to those of sorafenib, including vascular endothelial growth factor receptors (VEGFRs), platelet-derived growth factor receptor beta (PDGFR-β), fibroblast growth factor receptor 1 (FGFR1), cytokine receptor c-KIT, receptor tyrosine kinases (RET), and serine/threonine kinases c-Raf (Raf-1) and B-Raf [3, 4, 16, 17], a study reported improved median survival, by approximately 3 months, with regorafenib, compared to that with placebo after the failure of sorafenib [7]. These findings hint at new targets that can reverse drug resistance to sorafenib [16–18].

The PI3K/AKT pathway is highly activated in HCC. It is involved in the development and progression of HCC as it regulates numerous downstream molecules [19]. Consistent with the findings of our previous studies [20–23], AKT activation has been reported to contribute to sorafenib resistance in HCC [24–26]. Chronic exposure of HCC cells to sorafenib activates AKT to upregulate the expression of and/or activate its downstream factors [21]. Inhibition of AKT reversed drug resistance to sorafenib and restored sorafenib responsiveness in preclinical studies [20, 21, 24]. Moreover, the activation of the PI3K/AKT pathway is correlated with sorafenib resistance in HCC clinical studies and is specifically observed in the progenitor subclass of HCC [27, 28]. Although AKT inhibitors, as promising second-line treatment drugs, for HCC, have been thoroughly evaluated [29, 30], none of these have been clinically administered due to limited responses in HCC [3, 31]. We conducted an in-depth study of the mechanism of AKT activation during drug resistance to sorafenib and explored the potential therapeutics that could reverse this resistance.

Human kinesin family member 14 (KIF14), a member of the kinesin-3 superfamily of microtubule-dependent cytoskeletal motor proteins, is located on chromosome 1q32.1 and is involved in cytokinesis [32, 33]. The oncogene *KIF14* is activated in several cancers, including HCC [32–35]. Its suppression inhibits cell proliferation and promotes apoptosis by increasing p27 and decreasing cyclin D1 levels [35, 36]. Recently, KIF14 has been reported to regulate the PI3K/AKT pathway by upregulating the phosphorylation of AKT [33, 34, 36–38] and contribute to chemoresistance in breast cancer [38, 39]. Overexpression of transcription factor E26 transformation-specific sequence 1 (ETS1) is a common molecular event in the pathogenesis of HCC [40, 41] and is regulated by AKT [42, 43]. Moreover, ETS1 upregulates the expression of KIF14 [44] and is involved in drug resistance to sorafenib in HCC [45]. These findings suggest that the ETS1–KIF14 pathway may function downstream of AKT activation and represent a new therapeutic target to reverse drug resistance to sorafenib. We investigated whether the ETS1–KIF14 pathway is involved downstream of activated AKT and whether downregulating KIF14 enhanced the efficacy of sorafenib in sorafenib-resistant HCC by inhibiting AKT activation.

## RESULTS

### Analysis of target genes involved in drug resistance to sorafenib

Despite being the first molecular targeted drug for HCC, development of resistance to sorafenib has always remained a concern. Recently, regorafenib, another new molecular target drug, has been approved for the second-line treatment after sorafenib failure in patients with HCC [7]. To study the effects, mechanism(s), and downstream targets of regorafenib and to determine how it reverses sorafenib resistance, bioinformatics methods were used. First, the differentially expressed genes (DEGs) related to sorafenib and regorafenib were obtained from GSE89410 and DrugBank by comparing the treated and untreated samples. Next, the potential target genes of sorafenib and regorafenib from GSE89410 and DrugBank were integrated, and 699 and 727 target genes of sorafenib and regorafenib, respectively, were obtained. Only 280 target genes overlapped between the two sets of DEGs (Supplementary Figures 1, 2).

**Figure 1:**
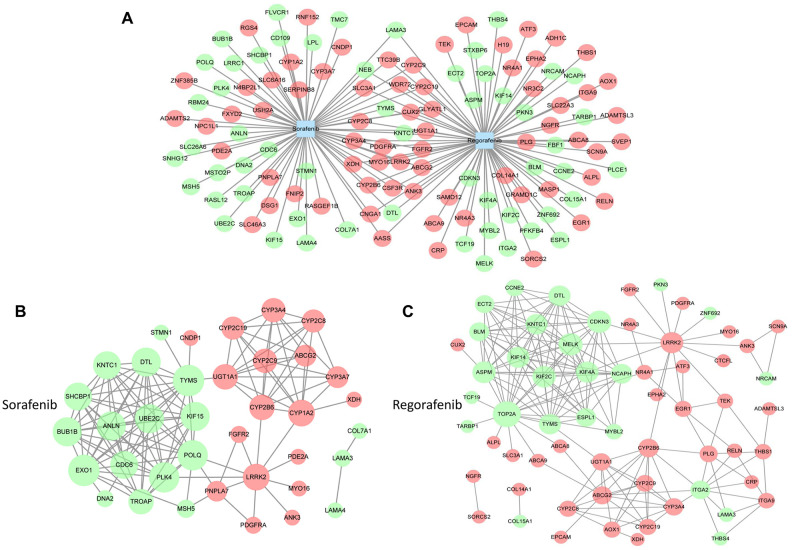
**Analysis of targets involved in drug resistance to sorafenib.** (**A**) The target genes of sorafenib and regorafenib were obtained from GSE89410 of the Gene Expression Omnibus (GEO) database (http://www.ncbi.nlm.nih.gov/geo/) and DrugBank database (https://www.drugbank.ca/). The differentially expressed genes (DEGs) of HCC were obtained from The Cancer Genome Atlas (TCGA, https://cancergenome.nih.gov/). Next, the overlapping target genes of sorafenib and regorafenib or the intersection genes in HCC were obtained by Venn analysis using an online tool (http://bioinformatics.psb.ugent.be/webtools/Venn/). (**B** and **C**) The network graphs were constructed using Cytoscape software. The protein–protein interaction (PPI) network of sorafenib (**B**) and regorafenib (**C**) was analyzed by STRING (https://string-db.org/).

To identify the DEGs in HCC, the expression of mRNAs in 371 HCC samples and 50 paired adjacent normal tissues obtained from The Cancer Genome Atlas (TCGA) dataset was analyzed. A total of 1,148 mRNAs were found to be aberrantly expressed in HCC tissues, including 474 downregulated mRNAs and 674 upregulated mRNAs. The identified DEGs in HCC were intersected with the potential target genes of sorafenib and regorafenib to identify the common genes. In total, 70 potential target genes of sorafenib and 80 potential target genes of regorafenib were related to HCC. Moreover, 26 genes targeted by both sorafenib and regorafenib were related to HCC, as determined by regulatory network analysis (Figure 1A, Supplementary Figure 3). The functional and pathway enrichment analysis of screened DEGs was performed using the Database for Annotation, Visualization and Integrated Discovery (DAVID). The top 10 enriched Kyoto Encyclopedia of Genes and Genomes (KEGG) pathways of sorafenib and regorafenib in HCC are shown in Supplementary Tables 1, 2. In particular, 15 potential target genes of regorafenib were mainly enriched in the PI3K/AKT signaling pathway. This finding is consistent with that of our previous study that demonstrated that activation of the PI3K/AKT signaling pathway was responsible for the acquired resistance to sorafenib and that inhibition of AKT reversed the resistance in HCC [20–23].

Next, to understand the link between the intersected genes, we constructed a protein–protein interaction (PPI) network based on the target genes of sorafenib and regorafenib in HCC. As shown in Figure 1B, 1C, the PPI network of sorafenib consisted of 37 protein nodes and 130 edges, whereas the regorafenib network consisted of 62 protein nodes and 181 edges. Among these nodes, 17 protein nodes were present in the intersection of sorafenib and regorafenib, and 82 nodes were used for survival curve analysis. In total, 38 genes related to the survival of patients with HCC (Supplementary Table 3). TOP2A, MELK, KIF2C, ASPM, KIF4A, and KIF14 were among the 10 highest degree target proteins of regorafenib that were related to the survival in HCC (Supplementary Tables 3, 4). The above results also indicate that certain targets of regorafenib are similar to those of sorafenib; however, more distinct targets exist that may represent the latent targets involved in the reversal of sorafenib resistance, especially those involved in the PI3K/AKT pathway and the single target of regorafenib related to survival.

### KIF14 is upregulated in sorafenib-resistant HCC cells

**Figure 2:**
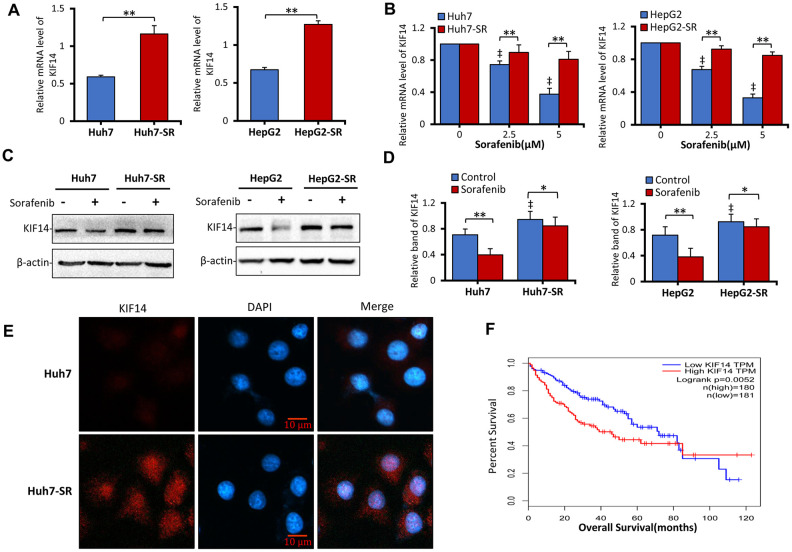
**KIF14 is upregulated in sorafenib-resistant HCC cells.** (**A** and **B**) Sorafenib-resistant Huh7-SR and HepG2-SR cells and the corresponding parent cells were incubated with 0, 2.5, or 5 μM sorafenib for 48 h. KIF14 mRNA levels were measured by quantitative reverse transcription-polymerase chain reaction (qRT-PCR) and normalized against glyceraldehyde 3-phosphate dehydrogenase (GAPDH). (**B**) The relative KIF14 mRNA levels of cells treated with 0 μM sorafenib were normalized to 1. (**C** and **D**) Sorafenib-resistant Huh7-SR and HepG2-SR cells and the corresponding parent cells were incubated with 0 or 5 μM sorafenib for 48 h. The protein expression profiles were detected by western blotting (**C**). The density of each band was normalized to that of β-actin (**D**). (**E**) Huh7-SR and Huh7 cells were stained with anti-KIF14 Ab (red) and DAPI (cellular nuclei, blue) and viewed with an inverted fluorescence microscope. (**F**) The KIF14 expression was used for survival analysis based on the TCGA database. The data represent three independent experiments. Scale bar = 10 μm. “*” Indicates P<0.05, and “**” indicates P<0.001; “‡” indicates P<0.001 versus untreated parent cells.

As described in our previous studies [20–22, 46], we established and evaluated two sorafenib-resistant cell lines by culturing human HCC Huh7 and HepG2 cells in gradually increasing concentrations of sorafenib and named them Huh7-SR and HepG2-SR, respectively. To investigate the function of latent genes in drug resistance to sorafenib in HCC, we first detected the mRNA expression of TOP2A, MELK, KIF2C, ASPM, KIF4A, and KIF14 in sorafenib-resistant Huh7-SR and HepG2-SR cells and compared it with that in the corresponding parent cells. As shown in Figure 2A and Supplementary Figure 4A, compared with the corresponding parent cells, only the expression of *KIF14* was upregulated among the latent genes in sorafenib-resistant Huh7-SR and HepG2-SR cells. Although sorafenib still exhibited antitumor activity in parent cells by inhibiting the expression of these genes and downregulating KIF14 in a concentration-dependent manner, its effect in downregulating the expression of KIF14 in sorafenib-resistant cells weakened markedly (Figure 2B and Supplementary Figure 4B).

Next, to investigate the protein expression of KIF14, sorafenib-resistant HCC cells and parent cells were incubated with 0 or 5 μM sorafenib for 48 h and subsequently subjected to western blotting. As shown in Figure 2C, 2D, compared with the corresponding parent cells, KIF14 was upregulated in sorafenib-resistant cells. Moreover, the upregulated KIF14 expression was confirmed in sorafenib-resistant Huh7-SR cells by immunofluorescence assay (Figure 2E). The high expression of KIF14 indicated poor OS in patients with HCC (Figure 2F). The above results demonstrated that the elevated expression of KIF14 was refractory to sorafenib-induced downregulation in sorafenib-resistant HCC cells.

### KIF14 silencing reverses acquired resistance to sorafenib in sorafenib-resistant HCC cells

**Figure 3:**
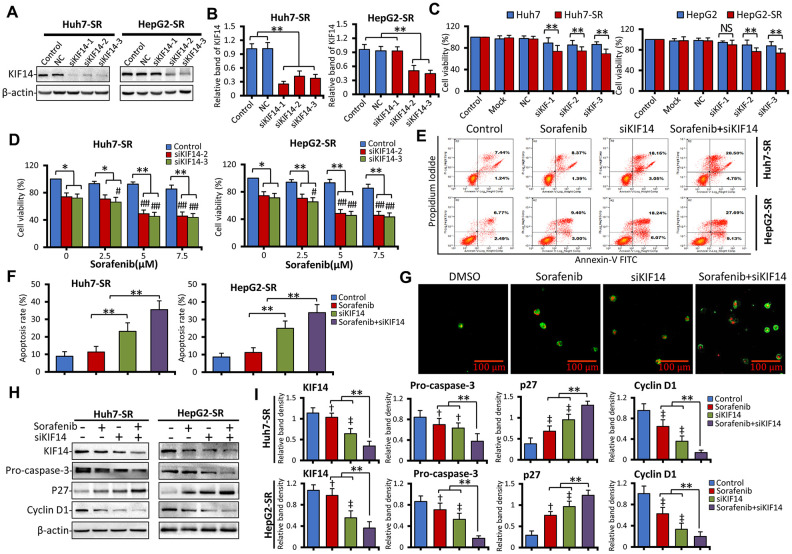
**Silencing of *KIF14* reverses acquired resistance to sorafenib in sorafenib-resistant HCC cells.** (**A** and **B**) Sorafenib-resistant Huh7-SR and HepG2-SR cells were transfected with siKIF14 or negative control (NC) for 48 h. The corresponding untransfected cells served as the control. The protein expression profiles were detected by western blotting (**A**). The density of each band was normalized to that of β-actin (**B**). (**C**) Huh7-SR and HepG2-SR cells and the corresponding parent cells were transfected with siKIF14 or NC for 48 h. The transfection reagents served as the mock control. The corresponding untransfected cells served as the control. Cell viability (%) of transfected cells was compared with that of the corresponding untreated cells. (**D**) Huh7-SR and HepG2-SR cells were transfected with control or siKIF14 for 24 h and subsequently incubated with increasing concentrations of sorafenib for 24 h. The cell viability (%) of transfected cells was compared with that of the corresponding untreated cells. (**E** and **G**) Huh7-SR and HepG2-SR cells were transfected with control or siKIF14 for 24 h and subsequently incubated with 0 or 5 μM sorafenib for 24 h. (**E** and **F**) Flow cytometry was used to detect apoptosis and measure the rate of apoptosis. (**G**) Representative images were acquired from Huh7-SR cells stained with annexin V–fluorescein isothiocyanate (FITC)/propidium iodide (PI) and viewed by microscopy. Green fluorescent membranes alone represented early-stage apoptotic cells, and green fluorescent membranes in combination with red fluorescent nuclei represented late-stage apoptotic cells. (**H** and **I**) Cells from (**E**) were subjected to western blotting to detect the protein expression profiles. The density of each band was normalized to that of β-actin. Data represent three independent experiments. Scale bar = 100 μm. NS, not significant. “**” Indicates P<0.001; “†” indicates P<0.05, and “‡” indicates P<0.001 versus untreated parent cells; # indicates P<0.05, and “##” indicates P<0.001 versus siKIF14 alone.

To further investigate the function of KIF14 in acquired resistance to sorafenib, we designed and synthesized three siRNAs to silence *KIF14* (Table 1). As shown in Figure 3A, 3B and Supplementary Figure 5, compared with the corresponding control, siKIF14 downregulated the KIF14 protein in Huh7-SR and HepG2-SR cells. Consistent with the downregulated protein expression of KIF14, siRNA-mediated silencing of *KIF14* reduced the cell viability. Moreover, sorafenib-resistant cells were more sensitive to a siKIF14-induced decrease in cell viability than the corresponding parent cells (Figure 3C). Considering the poor effects of siKIF14-1 in HepG2-SR cells in inhibiting KIF14 protein expression and reducing cell viability, siKIF14-2 and siKIF14-3 were used in subsequent experiments.

**Table 1:** siRNAs and their target genes used in the study.

**Genes**	**siRNA sequences**	
	Forward: 5’ to 3’	Reverse: 5’ to 3’
*KIF14-1*	CUCAGAGCAAGUUGGAUAUTT	AUAUCCAACUUGCUCUGAGTT
*KIF14-2*	GCCCGUUUAAUAGUCAACATT	UGUUGACUAUUAAACGGGCTT
*KIF14-3*	GCCAUCUGGAAGAGAUACUTT	AGUAUCUCUUCCAGAUGGCTT
*ETS1*	ACUUGCUACCAUCCCGUACTT	GUACGGGAUGGUAGCAAGUTT [52]
NC	UUCUCCGAACGUGUCACGUTT	ACGUGACACGUUCGGAGAATT

Abbreviations: ETS1, transcription factor E26 transformation-specific sequence 1; KIF14, kinesin family member 14; NC, negative control.

Consequently, we next analyzed the effect of siKIF14 in combination with sorafenib. As shown in Figure 3D, Huh7-SR and HepG2-SR cells were transfected with control or *KIF14* siRNAs for 24 h and subsequently incubated with increasing concentrations of sorafenib for another 24 h. Although sorafenib alone had a limited effect on reducing the cell viability in sorafenib-resistant cells—a drug resistance characteristic as previously described [20–22]—siKIF14 in combination with sorafenib reduced the cell viability. To investigate whether the silencing of *KIF14* synergized with sorafenib to reduce cell viability, the coefficient of drug interaction (CDI) was calculated, as described previously [47]. The CDIs of siKIF14 in combination with 5 and 10 μM sorafenib were less than 1, indicating marked synergistic effects (Supplementary Tables 5 and 6). The most optimized synergistic effect was exerted by siKIF14-3 in combination with 5 μM sorafenib, yielding CDIs of 0.69 and 0.66 for HepG2-SR and Huh7-SR cells, respectively. This combination was consequently used in subsequent experiments. Although the silencing of *KIF14* did not show the synergistic effect with sorafenib in parent cells (Supplementary Figure 6), this combination promoted apoptosis in sorafenib-resistant cells. As shown in Figure 3E, 3F, siKIF14 promoted apoptosis compared with the control or sorafenib alone. Further, compared with siKIF14 or sorafenib alone, a combination of siKIF14 and sorafenib promoted apoptosis. These results were also confirmed by detecting annexin V/propidium iodide (PI)-stained cells using confocal microscopy (Figure 3G). Furthermore, the silencing of *KIF14* synergized with sorafenib to downregulate pro-caspase 3 and cyclin D1 and upregulate p27 protein expression in sorafenib-resistant cells (Figure 3H, 3I), which are downstream effectors of KIF14 and targets of sorafenib, and regulate cell proliferation and apoptosis [35, 48–50]. These results indicated that the silencing of *KIF14* reversed acquired resistance to sorafenib in sorafenib-resistant cells.

### KIF14 silencing reverses acquired resistance to sorafenib by downregulating p-AKT

**Figure 4:**
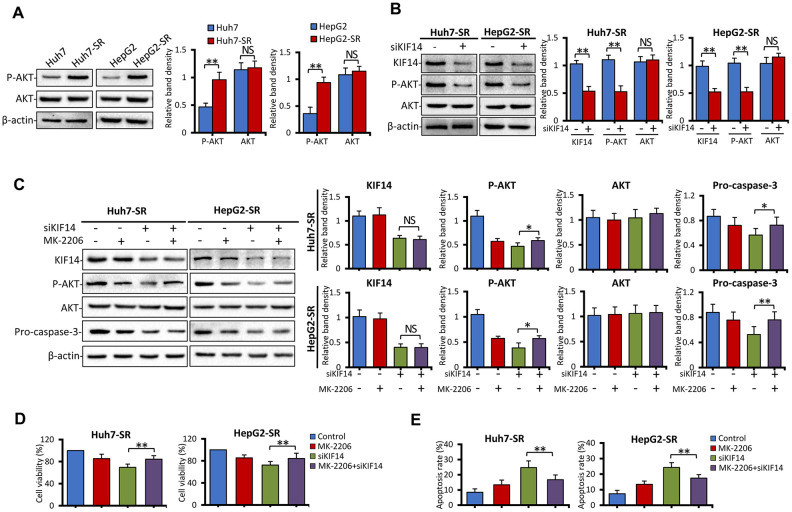
***KIF14* silencing reverses acquired resistance to sorafenib by downregulating p-AKT.** (**A**) The protein expression profiles of sorafenib-resistant Huh7-SR and HepG2-SR cells and the corresponding parent cells were detected by western blotting. Representative protein bands are shown. The density of each band was normalized to that of β-actin. (**B**) Huh7-SR and HepG2-SR cells were transfected with control or siKIF14 for 48 h. The protein expression profiles were detected by western blotting. The density of each band was normalized to that of β-actin. (**C**–**E**) Huh7-SR and HepG2-SR cells were pretreated with 0 or 10 μM MK-2206 for 24 h, transfected with control or siKIF14, and incubated in a medium containing MK-2206 for another 24 h. Subsequently, the cells were harvested for analysis. The protein expression profiles were detected by western blotting. The density of each band was normalized to that of β-actin (**C**). The cell viability (%) of treated cells was compared with that of the corresponding untreated cells (**D**). The percentages of apoptotic cells (%) were measured by flow cytometry (**E**). Data represent three independent experiments. NS, not significant. “*” Indicates P<0.05 and “**” indicates P<0.001.

As shown in our previous studies [21, 22], the activation of AKT, as evident from upregulated p-AKT, is responsible for the acquired resistance to sorafenib. Further, inhibition of AKT synergizes with sorafenib to reverse drug resistance. Therefore, in the current study, we next assessed the expression of AKT. As shown in Figure 4A, the expression of p-AKT was upregulated, whereas that of total AKT protein did not in sorafenib-resistant cells compared with the corresponding parent cells. These results indicated that the activation of AKT. in sorafenib-resistant cells occurred after its translational modification. Recently, KIF14 was reported to cause tumorigenesis in different cancers, including HCC, and activate AKT [33, 34, 36–38]. To further investigate whether upregulation of KIF14 could activate AKT, sorafenib-resistant cells were transfected with siKIF14 or control siRNA for 48 h, and subsequently harvested for western blotting. As shown in Figure 4B, the silencing of *KIF14* downregulated p-AKT but did not affect the total AKT protein levels. These results indicated that KIF14 regulated the activation of AKT via a posttranslational modification in sorafenib-resistant cells.

Consequently, 8-[4-(1-aminocyclobutyl)phenyl]-9-phenyl-[1,2,4]triazolo[3,4-f] [1,6]naphthyridin-3(2H)-one dihydrochloride (MK-2206), a highly selective non-ATP competitive allosteric inhibitor of AKT, was used. Sorafenib-resistant cells were pretreated with 0 or 10 μM MK-2206 for 24 h, transfected with control or siKIF14, and incubated in a medium containing MK-2206 for another 24 h. The cells were harvested for analysis. As shown in Figure 4C, the silencing of *KIF14* downregulated p-AKT, whereas downregulation of p-AKT by MK-2206 had no effect on the expression of KIF14. Moreover, siKIF14 or MK-2206 alone downregulated pro-caspase 3. However, when cells were pretreated with MK-2206 to inhibit the AKT pathway, the effect of *KIF14* silencing on the expression of pro-caspase 3 diminished while showing a similar inhibitory effect on the expression of KIF14 (Figure 4C). Furthermore, we investigated whether *KIF14* silencing inhibited proliferation and promoted apoptosis after pretreatment with MK-2206. As shown in Figure 4D, 4E, pretreatment with MK-2206 attenuated these effects. These results indicate that AKT functions downstream of KIF14 and that silencing of *KIF14* reverses the acquired resistance to sorafenib by downregulating p-AKT.

### KIF14 silencing blocks the AKT–ETS1–KIF14 positive feedback loop to reverse acquired resistance to sorafenib in HCC

**Figure 5:**
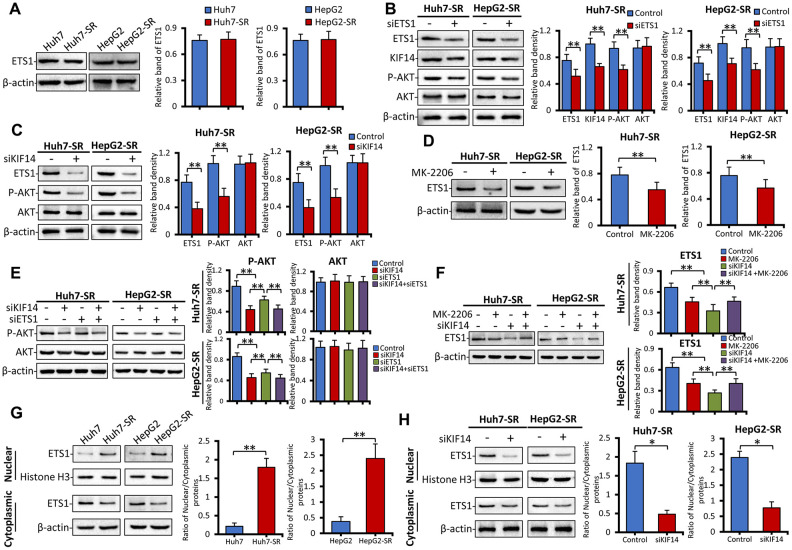
**The AKT–ETS1–KIF14 positive feedback loop is responsible for the activation of KIF14.** (**A**) The protein expression profiles ETS1 in sorafenib-resistant Huh7-SR and HepG2-SR cells and the corresponding parent cells were detected by western blotting. Representative protein bands are shown. The density of each band was normalized to that of β-actin. (**B**) Huh7-SR and HepG2-SR cells were transfected with control or siETS1 for 48 h. The protein expression profiles of ETS1, AKT, p-AKT, and KIF14 were detected by western blotting. The density of each band was normalized to that of β-actin. (**C**) Huh7-SR and HepG2-SR cells were transfected with control or siKIF14 for 48 h. The protein expression profiles of ETS1, AKT, and p-AKT were detected by western blotting. The density of each band was normalized to that of β-actin. (**D**) Huh7-SR and HepG2-SR cells were incubated with 0 or 10 μM MK-2206 for 48 h. The protein expression profile of ETS1 was detected by western blotting. The density of each band was normalized to that of β-actin. (**E**) Huh7-SR and HepG2-SR cells were transfected with control or siKIF14 for 24 h and subsequently transfected with control or siETS1 for another 24 h. The protein expression profiles of AKT and p-AKT were detected by western blotting. The density of each band was normalized to that of β-actin. (**F**) Huh7-SR and HepG2-SR cells were incubated with 0 or 10 μM MK-2206 for 24 h and subsequently transfected with control or siKIF14 for another 24 h. The protein expression profile of ETS1 was detected by western blotting. The density of each band was normalized to that of β-actin. (**G** and **H**) The nuclear and cytoplasmic protein expression profiles of ETS1 in sorafenib-resistant cells, and the corresponding parent cells from (**A**) were detected by western blotting (**G**). The nuclear and cytoplasmic protein expression profiles of ETS1 in Huh7-SR and HepG2-SR cells, transfected with control or siKIF14 from (**C**), were detected by western blotting (**H**). The band density of nuclear and cytoplasmic proteins was normalized to that of Histone H3 and β-actin, respectively. The ratio of nuclear/cytoplasmic protein was calculated. Data represent three independent experiments. “*” Indicates P<0.05 and “**” indicates P<0.001.

We next investigated the mechanism of KIF14 upregulation in sorafenib-resistant HCC cells. ETS1, a transcription factor and downstream molecule of the Raf/mitogen-activated protein kinase (MAPK)/extracellular signal-regulated kinase (ERK) signaling pathway and the PI3K/AKT pathway [42, 43, 51, 52], is downregulated by sorafenib [53] and is involved in drug resistance to sorafenib in HCC [45, 50]. Moreover, ETS1 upregulated the expression of KIF14 in glioma [44]. Therefore, in the current study, we investigated if ETS1 could activate KIF14 in sorafenib-resistant HCC. As shown in Figure 5A, the total protein expression of ETS1 was not different between parent and sorafenib-resistant cells. The silencing of *ETS1* downregulated the expression of KIF14 and p-AKT but did not affect the total AKT expression (Figure 5B). Silencing of *KIF14* inhibited the phosphorylation of AKT and simultaneously downregulated the expression of ETS1 (Figure 5C). Moreover, the expression of ETS1 was downregulated when MK-2206 was used to inhibit the AKT pathway (Figure 5D). To further explore the latent molecular mechanism, sorafenib-resistant cells were pretreated with siKIF14 and MK-2206, and subsequently, the protein levels of ETS1 and AKT were detected by western blotting. As shown in Figure 5E, the cells were transfected with control or *KIF14* siRNA for 24 h and next transfected with control or *ETS1* siRNA for another 24 h. The cells were finally harvested for analysis. The silencing of either *KIF14* or *ETS1* downregulated the expression of p-AKT; however, it did not affect the expression of AKT. However, following the siRNA-mediated knockdown of *KIF14*, siETS1 did not further amplify the inhibitory effect of siKIF14 on p-AKT. These results indicated that KIF14 functions downstream of ETS1 and that silencing of *ETS1* downregulates the expression of p-AKT by inhibiting the expression of KIF14. Moreover, pre-treatment of sorafenib-resistant cells with MK-2206 to inhibit p-AKT abrogated the siKIF14-induced inhibition of ETS1 (Figure 5F). Compared with parent HCC cells, ETS1 translocated from the cytoplasm to the nucleus in sorafenib-resistant cells (Figure 5G). The silencing of *KIF14* downregulated the expression of nuclear ETS1 protein and inhibited its translocation (Figure 5H). These results indicate that ETS1 functions downstream of KIF14 and that silencing of *KIF14* inhibits ETS1 by downregulating the expression of p-AKT. Altogether, the AKT–ETS1–KIF14 positive feedback loop is at least partially responsible for the upregulation of KIF14 and further AKT activation during drug resistance to sorafenib in HCC.

**Figure 6:**
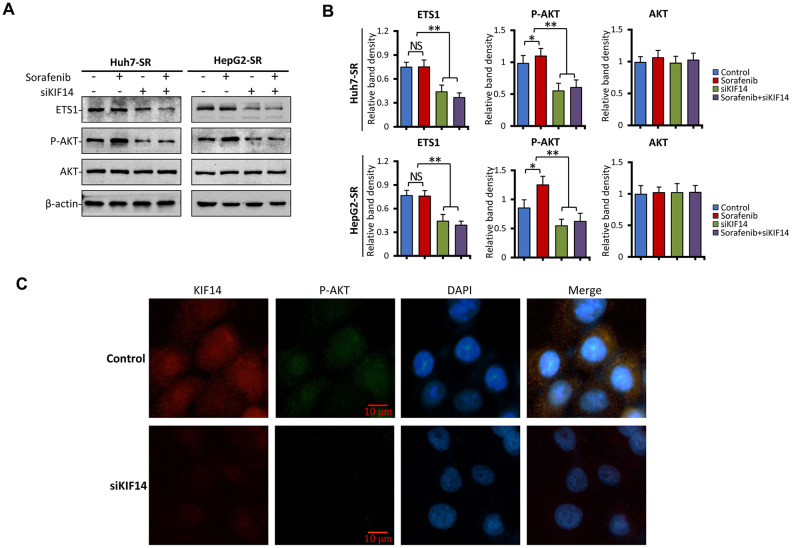
***KIF14* silencing inhibits the AKT–ETS1–KIF14 positive feedback loop to reverse acquired resistance to sorafenib in HCC.** (**A** and **B**) Sorafenib-resistant Huh7-SR and HepG2-SR cells were transfected with control or siKIF14 for 24 h and subsequently incubated with 0 or 5 μM sorafenib for 24 h. The protein expression profiles of ETS1, AKT, and p-AKT were detected by western blotting (**A**). The density of each band was normalized to that of β-actin (**B**). (**C**) Control or siKIF14-transfected Huh7-SR cells were stained with anti-KIF14 Ab (red), anti-p-Akt Ab (green), and DAPI (cellular nuclei, blue) and viewed with an inverted fluorescence microscope. Data represent three independent experiments. Scale bar = 10 μm. NS, not significant. “*” Indicates P<0.05 and “**” indicates P<0.001.

To explore the molecular mechanism of *KIF14* silencing in reversing acquired resistance to sorafenib in HCC, sorafenib-resistant cells were transfected with control or *KIF14* siRNA for 24 h, incubated with 0 or 5 μM sorafenib for another 24 h, and finally subjected to western blotting. As shown in Figure 6A, 6B and consistent with the results of our previous studies [21, 22], sorafenib upregulated the expression of p-AKT in sorafenib-resistant cells but did not affect the expression of AKT. The silencing of *KIF14* inhibited the activation of AKT and simultaneously downregulated the expression of ETS1, whereas sorafenib alone had no effect on the expression of ETS1. A combination of siKIF14 and sorafenib suppressed sorafenib-induced AKT activation and simultaneously downregulated the expression of ETS1. These results were further confirmed by immunofluorescence assay. Sorafenib-resistant cells showed upregulated expression of KIF14 and p-AKT. The silencing of *KIF14* simultaneously downregulated the expression of KIF14 and p-AKT (Figure 6C). These results indicate that the silencing of *KIF14* inhibited the AKT–ETS1–KIF14 positive feedback loop to reverse acquired resistance to sorafenib in HCC.

### KIF14 silencing synergizes with sorafenib to suppress subcutaneous tumors formed from sorafenib-resistant cells *in vivo*

**Figure 7:**
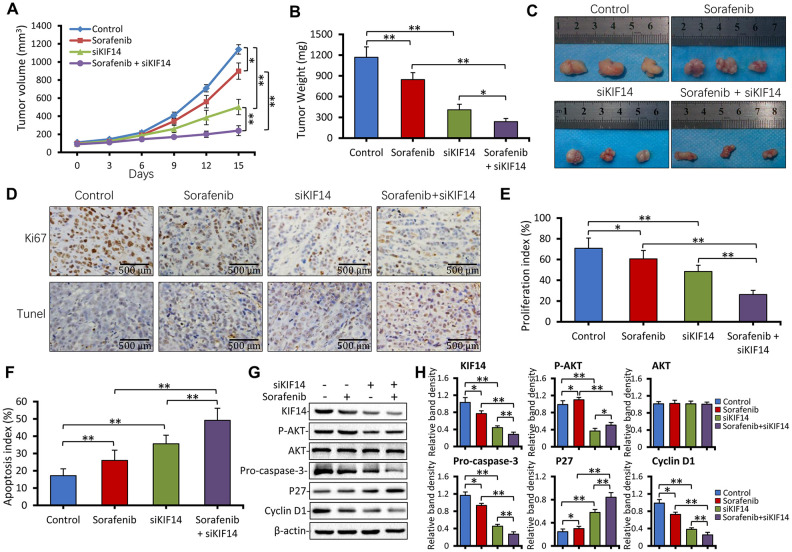
***KIF14* silencing synergizes with sorafenib to suppress subcutaneous tumors formed from sorafenib-resistant cells *in vivo*.** (**A**) Subcutaneous tumors were established in mice that received different treatments for 15 days as described in the Materials and Methods section. The volume (mm^3^) of tumors was recorded. (**B** and **C**) Tumors harvested at the end of experiments were weighed (B) and photographed (**C**). (**D**) Representative images of tumor sections stained with anti-Ki-67 Ab (top) and subjected to TUNEL assay (bottom; magnification, ×200). (**E**) The proliferation index (%) and (**F**) apoptosis index (%) were quantified. (**G** and **H**) Western blotting of lysates of tumors harvested from (**A**) at the end of experiments (**G**). The density of each band was normalized to that of β-actin (**H**). Scale bar = 500 μm. “*” Indicates P<0.05 and “**” indicates P<0.001.

As described in the Materials and Methods section here and in our previous studies [20, 21, 46], sorafenib- resistant Huh7-SR cells were subcutaneously inoculated into nude mice. The sorafenib-resistant ability of injected Huh7-SR cells was maintained by orally administering the drug daily to mice at a dose of 10 mg/kg. When the volume of tumors reached approximately 100 mm^3^, the mice were randomly assigned to four treatments. As shown in Figure 7A, the tumors were refractory to sorafenib because sorafenib-treated tumors were only slightly smaller than the control tumors (1138.3 ± 52.4 mm^3^ vs*.* 898.7 ± 89.2 mm^3^ in volume) 15 days after the commencement of treatments. However, intratumoral injection of siKIF14 alone reduced the size of tumors (501.3 ± 85.6 mm^3^ in volume) by 55.9%. Compared with control tumors, the combination of siKIF14 and sorafenib further reduced the tumor size (240.8 ± 54.9 mm^3^ in volume) by 78.8% at the end of experiments. Moreover, compared to the treatment with siKIF14 or sorafenib alone, siKIF14 synergized with sorafenib to reduce the tumor volumes (Figure 7A). The results were supported by the tumor weight (Figure 7B) and tumor size (Figure 7C) calculated at the end of the experiments.

We used Ki-67 immunohistochemistry and *in situ* terminal deoxynucleotidyl transferase dUTP nick end labeling (TUNEL) staining to detect cell proliferation and apoptosis, respectively. As shown in Figure 7D to 7F, sorafenib alone exerted a weak effect on cell proliferation inhibition and apoptosis promotion. However, siKIF14 alone exhibited a stronger proliferation inhibitory and proapoptotic activity than sorafenib alone in subcutaneous tumors. Moreover, the combination of siKIF14 and sorafenib showed stronger proliferation inhibitory and proapoptotic activity than siKIF14 or sorafenib alone (Figure 7E, 7F). The expression of downstream proteins was analyzed by western blotting (Figure 7G). Consistent with the *in vitro* results, sorafenib activated AKT, whereas siKIF14 inactivated AKT in subcutaneous tumors. The siKIF14 and sorafenib combination inhibited sorafenib-induced activation of AKT, downregulated the expression of pro-caspase-3 and cyclin D1, and upregulated the expression of p27 (Figure 7H).

## DISCUSSION

**Figure 8:**
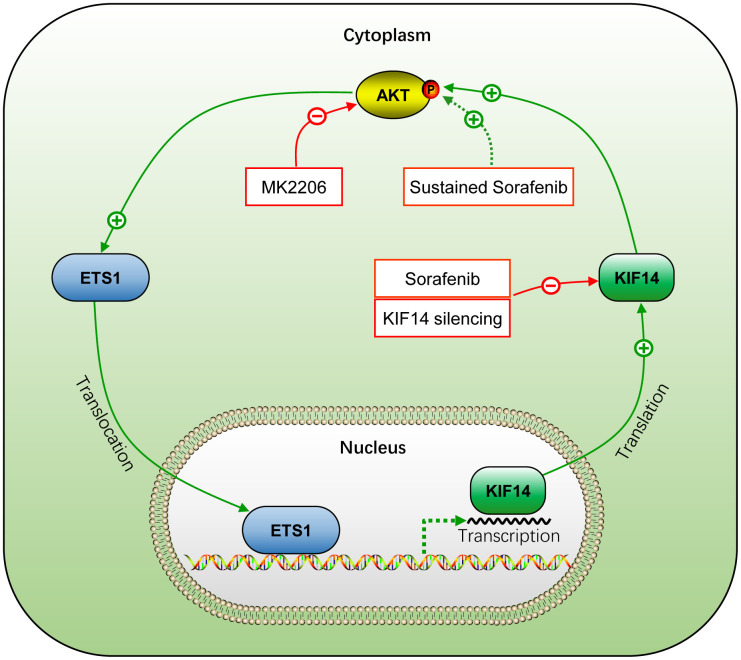
**Schematic representation of the activation of AKT–ETS1–KIF14 positive feedback loop during acquired resistance to sorafenib in HCC.** +, positive regulation or activation; −, negative regulation or blockade; p, regulation by phosphorylation. The solid line indicates direct interaction, whereas the dotted line indicates indirect interaction.

Although extensive studies have been conducted in the past few decades to enhance the efficacy of anticancer drugs by overcoming chemoresistance, it remains a major clinical challenge in HCC treatment [3]. Sorafenib is the first approved molecular targeted drug for HCC [4, 5]. As previously reported [20–22, 54], drug resistance to sorafenib is characterized by a low and partial response rate and reduced survival benefits and poses a serious concern due to a shortage of effective systemic treatments for HCC [5, 9]. The present study demonstrates that activation of KIF14 contributes to the resistance of HCC to sorafenib. Moreover, blocking KIF14 enhances the efficacy of sorafenib to combat HCC by inhibiting sorafenib-induced AKT activation to promote apoptosis and inhibit proliferation. Further, the activation of KIF14 is at least partially attributed to the AKT–ETS1–KIF14 positive feedback loop. The silencing of *KIF14* inhibits the AKT activation and simultaneously downregulates the expression of ETS1 by blocking the AKT–ETS1–KIF14 pathway (Figure 8).

Sorafenib and regorafenib are currently principal molecular targeted drugs for HCC. They represent the standard first-line treatment at initial diagnosis and second-line treatment after sorafenib failure in HCC, respectively [5, 7]. Although both these drugs have similar targets, regorafenib prolongs the median survival of patients by approximately another 3 months after sorafenib fails [5, 7]. Moreover, regorafenib has more potent pharmacological activity in preclinical studies [16] and is more effective at inhibiting angiogenesis than sorafenib [17]. However, more new effective targets, such as tyrosine kinase with immunoglobulin and epidermal growth factor homology domain 2 (TIE2), which are involved in the antitumor activity of regorafenib, are being uncovered [55, 56]. These results indicate that the action of regorafenib could be different from that of sorafenib and that a latent new mechanism of combating drug resistance to sorafenib may exist among the effective targets of regorafenib [3]. Here, we used bioinformatics methods to analyze the differentially expressed genes (DEGs) of sorafenib and regorafenib in HCC. *KIF14* was identified and verified as an oncogene involved in acquired resistance to sorafenib in HCC.

KIF14 was initially identified as a cytokinesis regulator. It is located at the central spindle and the midbody, where it exerts multiple biological activities, such as cargo-containing vesicle transport, mitotic spindle formation, chromosome segregation, and midbody formation [38]. Recently, KIF14 has been reported as a prognostic biomarker and oncogene in certain carcinomas, including breast cancer, colorectal cancer, gastric cancer, HCC, lung cancer, ovarian cancer, retinoblastoma, and glioblastoma [32, 34–36, 38, 57]. *KIF14* is a DEG in HCC, and its high expression indicates poor OS in patients with HCC, especially during the first 80 months after the initial diagnosis (Figure 2F). Although no tumorigenesis was observed in a KIF14-overexpressing transgenic mouse model, enhanced proliferation was observed in multiple tissues including the liver [57]. This finding suggested that KIF14 rarely functions as an initiating oncogene but as an accelerator of tumor development in response to other oncogenic insults [57]. We previously showed that sustained exposure to sorafenib activated AKT, thus stimulating drug resistance to sorafenib in HCC cells [20–22]. Recently, silencing of *KIF14* downregulated p-AKT by its direct interaction at the plasma membrane [33, 34, 36–38] and reversed drug resistance to docetaxel but not to doxorubicin, carboplatin, or gemcitabine in breast cancer, indicating the function of KIF14 in altering drug resistance by effectively targeting antitumor drugs [38]. Herein, we demonstrated that the expression of KIF14 was upregulated in sorafenib-resistant HCC cells, and its silencing inhibited the activation of AKT by downregulating p-AKT, but not AKT, to reverse the acquired resistance to sorafenib.

Activation of the PI3K/AKT pathway is a common phenomenon in the development and progression of HCC. A positive rate was reported in 92.3% of HCC tissues [19, 58]. We previously found that activation of AKT induced drug resistance to sorafenib in HCC [21, 22], which is confirmed by current and other studies [24–26]. Sorafenib-resistant HCC cells were established by sustained treatment of initially responsive HCC parent cells with sorafenib. These cells showed sorafenib resistance accompanied by AKT activation [21] and inhibited AKT-reversed drug resistance to sorafenib by regulating downstream molecules involved in apoptosis, autophagy, endoplasmic reticulum, and EMT [20–22, 54]. We showed that AKT was activated in sorafenib-resistant HCC cells, and blocking it with a small molecule inhibitor, such as MK-2206, induced cell apoptosis and inhibited cell proliferation. Unfortunately, while AKT serves as a promising target for reversing drug resistance to sorafenib in preclinical studies, its inhibitors have poor clinical accessibility due to limited responses [3, 19].

ETS1, an oncogenic transcription factor and a downstream target of the PI3K/AKT pathway, contributes to the development and progression of several cancers, including HCC [43, 59, 60]. Moreover, ETS1 is considered an angiogenesis regulator and is upregulated in an AKT-dependent manner in primary endothelial cells [59, 60]. Both ETS1 and AKT are regulated by TIE2 in endothelial cells; however, this was not investigated in the present study [61]. TIE2 contains binding sites for ETS1 in its promoter region [62] and controls angiogenesis. It is an effective target of regorafenib; however, it is not inhibited by sorafenib, an established angiogenesis inhibitor [16]. ETS1 is upregulated in HCC [41, 45] and is involved in drug resistance to sorafenib in HCC [45, 50]. Furthermore, its overexpression accelerates the metabolic clearance of sorafenib to induce resistance to the drug in HCC cells, whereas its blocking enhances the antitumor activity of sorafenib [45]. Here, we demonstrated that inhibiting AKT by MK-2206 downregulated the expression of ETS1. A recent study showed KIF14 as the downstream molecule of ETS1 [44]. ETS1 promotes angiogenesis by upregulating the expression of KIF14 in glioma [44]. Herein, we showed that the silencing of *ETS1* simultaneously downregulated the expression of KIF14 and inhibited the activation of AKT. The silencing of *KIF14* simultaneously inhibited the activation of AKT and downregulated the expression of ETS1. Although we could not detect the type of interaction (direct or indirect) between these molecules, re-expression of silenced protein will strengthen the above results. However, inhibiting AKT did not inhibit KIF14, which may explain the poor effect of AKT inhibitors in clinical trials [3, 31]. In the present study, the expression of ETS1 did not differ between sorafenib-resistant and parent HCC cells. Generally, transcription factors are translated in the cytoplasm and regulate transcription of genes in the nucleus by binding to a specific DNA sequence using their DNA-binding domain. ETS1 is located both in the nucleus and cytoplasm. A recent study reported the nuclear exclusion of ETS1 via nucleus-to-cytoplasm translocation [63]. In particular, the overexpression of nuclear ETS1 was related to drug resistance to vemurafenib in melanoma [64, 65]. Another study showed the migration of ETS1 into the nucleus to upregulate the expression of KIF14 [44]. Similarly, here, we showed that ETS1 translocated from the cytoplasm to the nucleus in sorafenib-resistant cells and silencing of *KIF14* inhibited this translocation. These results demonstrated that not only the expression but also the location of ETS1 could be involved in controlling transcription and consequent biological effects.

In summary, the present study demonstrates that sustained exposure to sorafenib activates AKT, which subsequently upregulates the expression of KIF14 by elevating the expression of transcription factor ETS1 in sorafenib-resistant HCC cells. Upregulation of KIF14 contributes to the resistance of HCC to sorafenib. Its blocking synergizes with sorafenib to reverse the acquired resistance to the drug by inhibiting AKT activation and downregulating the expression of ETS1 by blocking the AKT–ETS1–KIF14 positive feedback loop (Figure 8). These findings warrant further investigation of KIF14 as a second-line therapeutic target after sorafenib failure or in combination with sorafenib to strengthen the antitumor effects in HCC.

## MATERIALS AND METHODS

### Target gene profiles of sorafenib and regorafenib

The expression profiles of target genes of sorafenib and regorafenib were obtained from the Gene Expression Omnibus (GEO) database (http://www.ncbi.nlm.nih.gov/geo/) under the accession number GSE89410 [66]. It consisted of data obtained from patient-derived pluripotent stem cells treated with 1 μM sorafenib, 1 μM regorafenib, and untreated control cells. Furthermore, the target genes of sorafenib and regorafenib were obtained from the DrugBank database (https://www.drugbank.ca/, version 4.0) [67]. The expression profiles of DEGs in HCC were downloaded from TCGA (https://cancergenome.nih.gov/) [68]. The intersection of target genes of sorafenib or regorafenib and DEGs of HCC was analyzed by Venn analysis using the online tool (http://bioinformatics.psb.ugent.be/webtools/Venn/). The Kyoto Encyclopedia of Genes and Genomes (KEGG) pathway enrichment analysis was performed using DAVID (https://david.ncifcrf.gov/, version 6.8) [69], and the PPI network analysis was performed using the online tool STRING (https://string-db.org/, version 10.5) [70]. The network graphs were constructed using Cytoscape software (version 3.5.1) [71]. The genes from the protein nodes of the PPI network were used for Kaplan–Meier survival analysis using the KEGG database.

### Cells, antibodies, and reagents

Human HCC Huh7 cell line was obtained from the Cell Bank of Type Culture Collection of the Chinese Academy of Sciences (Shanghai, China), and the HepG2 cell line was obtained from the American Type Culture Collection (ATCC; Manassas, VA, USA). The corresponding sorafenib-resistant cells, named Huh7-SR and HepG2-SR, were established by culturing the parent cell lines in gradually increased concentrations of sorafenib, as previously described [20–22, 46]. Cells were routinely cultured at 37°C in Dulbecco’s modified Eagle’s medium (DMEM) (Gibco BRL; Grand Island, NY, USA) supplemented with 10% fetal bovine serum (ExCellBio, Shanghai, China) under 5% CO_2_ in an incubator. The primary antibody (Ab) against KIF14 was purchased from Abcam (Cambridge, England). Abs against AKT, p-AKT (Ser473), caspase-3, p27, cyclin D1, and ETS1 were purchased from Cell Signaling Technology (Danvers, USA). The anti-β-actin, anti-Ki-67, and secondary Abs were purchased from Beijing Zhongshan Golden Bridge Biotechnology Co., Ltd. (Beijing, China). Sorafenib was purchased from Jinan Trio Pharmatech Co., Ltd. (Jinan, China). MK-2206 was purchased from Shanghai Biochempartner Co., Ltd. (Shanghai, China). The Cell Counting Kit-8 (CCK-8) was purchased from Dojindo Laboratories (Mashikimachi, Japan). The annexin V-fluorescein isothiocyanate (FITC)/propidium iodide (PI) apoptosis detection kit was obtained from BD Biosciences. The TUNEL assay agent was purchased from Roche.

### Quantitative reverse transcription-polymerase chain reaction

Huh7, HepG2, or the corresponding sorafenib-resistant cells in the logarithmic phase were incubated with 0, 2.5, or 5 μM sorafenib for 48 h, and total RNA was extracted using TRIzol (GENEWIZ Biotechnology Co., Ltd., Suzhou, China) according to the manufacturer’s instructions. The primers used to amplify human genes are shown in Table 2 and Supplementary Table 7. The methods have been described in detail previously [20, 46]. Briefly, the reverse transcription products obtained from the total RNA were loaded onto the TaqMan array for quantitative reverse transcription-polymerase chain reaction (qRT-PCR) using an Mx3000P real-time PCR system (Stratagene, USA). The specificity of amplification was confirmed by the melting curves. Relative mRNA levels of genes were calculated using the C_t_ values and normalized against that of glyceraldehyde 3-phosphate dehydrogenase (GAPDH), according to the equation: 2^−ΔCt^[ΔCt = Ct target gene−Ct GAPDH]. The experiments were repeated thrice, and the average results were calculated.

**Table 2:** Primers used for RT-PCR in the study.

**Genes**	**Gene ID**	**Primers (5’ to 3’)**
*KIF14*	9928	Forward: TGCCCCCAGTAGAGCAAAT
		Reverse: ACTCAGGGAAGCAATGGGTG
*ETS1*	2113	Forward: GTCGTGGTAAACTCGG
		Reverse: CAGCAGGAATGACAGG
*GAPDH*	2597	Forward: AAGAAGGTGGTGAAGCAGGC
		Reverse: TCCACCACCCAGT TGCTGTA

Abbreviations: ETS1, transcription factor E26 transformation-specific sequence 1; GAPDH, glyceraldehyde 3-phosphate dehydrogenase; KIF14, kinesin family member 14.

### Western blotting

The method for western blotting has been described previously [20, 22, 54]. Briefly, the total cellular proteins were extracted by cell lysis buffer (Beyotime Biotechnology; Beijing, China). The cellular, nuclear, and cytoplasmic proteins were extracted using a Nuclear and Cytoplasmic Protein Extraction Kit (BioTeke Corporation; Beijing, China) according to the manufacturer’s instructions. The protein concentration of cell lysates was determined using the protein assay kit (Bio-Rad; Richmond, CA, USA). The lysates were boiled in the sample buffer for 5 min. Next, 30 μg of protein was subjected to sodium dodecyl sulfate (SDS)-polyacrylamide gel electrophoresis, and the resolved proteins were transferred onto polyvinylidene difluoride (PVDF) membranes. The membranes were blocked with 5% skim milk in TBST (Tris-buffered saline with 0.1% Tween 20) and subsequently incubated with primary antibodies against KIF14, AKT, p-AKT, pro-caspase-3, p27, Cyclin D1, ETS1, and β-actin overnight at 4°C. After washing five times with TBST, the membranes were incubated with alkaline phosphatase-conjugated secondary Ab. The antibody–antigen complexes were observed by placing the membranes in a gel-imaging system and adding 200 μL/membrane of enhanced chemiluminescence (ECL) plus detection reagent (Pierce Chemical; Rockford, IL, USA). β-actin was used as the loading control. All experiments were repeated thrice.

### Immunofluorescence assay

Huh7 and HepG2 cells or Huh7-SR cells were transfected with control or *KIF14* siRNA for 48 h. Next, the cells were fixed with 4% paraformaldehyde and permeabilized in 0.5% Triton X-100. The cells were blocked with 5% bovine serum albumin (BSA) for 1 h at 37°C. Subsequently, the cells were incubated with anti-KIF14 and/or anti-AKT primary Abs overnight at 4°C, followed by incubation with fluorochrome-conjugated secondary antibody diluted in antibody dilution buffer for 1 h at room temperature in the dark. The DNA was stained using DAPI. Images of immunostained cells were acquired using an inverted fluorescence microscope.

### siRNA transfection

All siRNA duplexes were synthesized by Gemma Pharmaceutical Technology Co., Ltd. (Shanghai, China). Their sequences and corresponding target genes are shown in Table 1. The siRNAs targeting *KIF14* and *ETS1* were named siKIF14 and siETS1, respectively, and the negative control siRNA was called NC. The siRNA transfection methods have been previously described in detail [20–22, 72]. In brief, Huh7, HepG2, Huh7-SR, and HepG2-SR cells (5 × 10^5^ cells per well) in the logarithmic phase were cultured in 6-well plates for 24 h. After the cells had grown to 70% to 90% confluence, Lipofectamine 3000 (Thermo Fisher Scientific; Massachusetts, USA) and siKIF14, siETS1, or NC were diluted with Opti-MEM medium according to the manufacturer’s instructions. Next, the diluted siRNA was added to diluted Lipofectamine 3000 in equal volumes to obtain 50 nM as the final working concentration of siKIF14, siETS1, and NC. HCC cells were transfected with the corresponding siRNAs for 24 to 48 h and subsequently subjected to different assays. To verify the efficacy of siRNA, the cells were lysed, and the expression of related proteins was analyzed by western blotting. The experiments were repeated thrice.

### Cell viability assay

Cell viability was analyzed using the CCK-8 assay, which has been described previously [22]. In brief, Huh7, HepG2, or the corresponding sorafenib-resistant cells were plated in 96-well culture plates at a density of 3 × 10^3^ cells/well overnight, and transfected with siKIF14 and/or incubated with 10 μM MK-2206 or gradually increasing concentrations of sorafenib for 24 to 48 h. The culture medium was replaced with fresh medium containing 10 μL/well of CCK-8 solution for 2 h at 37°C, and the optical density (OD) at 450 nm was measured. Cell viability (%) was calculated according to the formula: (experiment OD value−blank OD value)/(control OD value−blank OD value) × 100%. The experiments were repeated thrice, and the average results were calculated.

### Detection of cell apoptosis *in vitro*

Cell apoptosis *in vitro* was detected by annexin V–FITC/PI staining and flow cytometry, as previously described [21–23]. Briefly, HCC cells were transfected with siKIF14 and/or incubated with 5 μM sorafenib or 10 μM MK-2206 for 24 to 48 h, and subsequently harvested. HCC cells (1 × 10^5^) were resuspended in 100 μL of binding buffer, and subsequently, 5 μL of annexin V and 5 μL of PI were added and incubated in the dark at room temperature for 15 min. Next, the apoptosis rate (%) was measured using the Beckman Coulter Epics Altra II cytometer (Beckman Coulter; California, USA). The experiments were repeated thrice, and the average results were calculated.

### Animal experiments

Male BALB/c-nu/nu immunodeficient mice (6 to 8 weeks old), obtained from Shanghai Laboratory Animal Center (SLAC) Laboratory Animal Co., Ltd. (Shanghai, China), were raised in specific pathogen-free (SPF) level mobile animal breeding rooms at the Fourth Affiliated Hospital of Harbin Medical University. The animal experiments were approved by the Animal Ethics Committee of Harbin Medical University (permit no. SYXK20020009) in compliance with the Experimental Animal Regulations by the National Science and Technology Commission, China. The protocol has been described previously [20, 21, 46, 54]. Briefly, Huh7-SR cells in the logarithmic phase were harvested and counted and subsequently diluted with serum-free DMEM medium. A total of 5 × 10^6^ cells were subcutaneously injected into the back of mice for tumor formation. To maintain the sorafenib-resistant ability of Huh7-SR cells, the drug was dissolved in a solvent containing chromophore (Sigma-Aldrich), 95% ethanol, and water in a volume ratio of 1:1:6 and was administered to mice at an oral dose of 10 mg/kg daily. Subcutaneous tumors were measured by a Vernier caliper every 3 days. The tumor volume was calculated according to the longest and shortest vertical diameters using the following formula: π/6 × a^2^ × b, where “a” represents the short axis, and “b” represents the long axis. When the tumor volume reached approximately 100 mm^3^, the mice were randomly assigned to four treatment groups (n = 6/group): control, sorafenib, siKIF14, and sorafenib + siKIF14. Sorafenib was administered to mice in the sorafenib and sorafenib + siKIF14 groups by gavage at a dose of 30 mg/kg daily. A total of 250 pmol siKIF14 was intratumorally injected by mixing with Lipofectamine 3000 at a concentration of 5 pmol/μL and injected once every 3 days, five times, in mice in the siKIF14 and sorafenib + siKIF14 groups. Mice in the sorafenib group simultaneously received an intratumoral injection of NC, and those in the control group received oral vehicle and an intratumoral injection of NC. The tumor volumes were measured every 3 days. The tumors were harvested at the end of the experiments.

### Immunohistochemistry, *in situ* detection of apoptotic cells, quantification of Ki-67 proliferation index

All these methods have been described in detail elsewhere [21, 54, 72].

### Statistical analysis

The data are expressed as mean values ± standard deviations (SDs). Statistical analysis was performed using the SPSS 20.0 statistical software (SPSS 224 Inc., IL, USA). Comparisons were made using one-way analysis of variance (ANOVA) followed by Dunnett’s *t*-test. A P<0.05 was considered significant.

## Supplementary Material

Supplementary Figures

Supplementary Tables

## References

[1] Bray F, Ferlay J, Soerjomataram I, Siegel RL, Torre LA, Jemal A. Global cancer statistics 2018: GLOBOCAN estimates of incidence and mortality worldwide for 36 cancers in 185 countries. CA Cancer J Clin. 2018; 68:394–424. 10.3322/caac.21492 30207593

[2] Villanueva A. Hepatocellular carcinoma. N Engl J Med. 2019; 380:1450–62. 10.1056/NEJMra1713263 30970190

[3] Llovet JM, Montal R, Sia D, Finn RS. Molecular therapies and precision medicine for hepatocellular carcinoma. Nat Rev Clin Oncol. 2018; 15:599–616. 10.1038/s41571-018-0073-4 30061739 PMC12452113

[4] Cheng AL, Kang YK, Chen Z, Tsao CJ, Qin S, Kim JS, Luo R, Feng J, Ye S, Yang TS, Xu J, Sun Y, Liang H, et al. Efficacy and safety of sorafenib in patients in the Asia-Pacific region with advanced hepatocellular carcinoma: a phase III randomised, double-blind, placebo-controlled trial. Lancet Oncol. 2009; 10:25–34. 10.1016/S1470-2045(08)70285-7 19095497

[5] Llovet JM, Ricci S, Mazzaferro V, Hilgard P, Gane E, Blanc JF, de Oliveira AC, Santoro A, Raoul JL, Forner A, Schwartz M, Porta C, Zeuzem S, et al, and SHARP Investigators Study Group. Sorafenib in advanced hepatocellular carcinoma. N Engl J Med. 2008; 359:378–90. 10.1056/NEJMoa0708857 18650514

[6] Kudo M, Finn RS, Qin S, Han KH, Ikeda K, Piscaglia F, Baron A, Park JW, Han G, Jassem J, Blanc JF, Vogel A, Komov D, et al. Lenvatinib versus sorafenib in first-line treatment of patients with unresectable hepatocellular carcinoma: a randomised phase 3 non-inferiority trial. Lancet. 2018; 391:1163–73. 10.1016/S0140-6736(18)30207-1 29433850

[7] Bruix J, Qin S, Merle P, Granito A, Huang YH, Bodoky G, Pracht M, Yokosuka O, Rosmorduc O, Breder V, Gerolami R, Masi G, Ross PJ, et al, and RESORCE Investigators. Regorafenib for patients with hepatocellular carcinoma who progressed on sorafenib treatment (RESORCE): a randomised, double-blind, placebo-controlled, phase 3 trial. Lancet. 2017; 389:56–66. 10.1016/S0140-6736(16)32453-9 27932229

[8] Abou-Alfa GK, Meyer T, Cheng AL, El-Khoueiry AB, Rimassa L, Ryoo BY, Cicin I, Merle P, Chen Y, Park JW, Blanc JF, Bolondi L, Klümpen HJ, et al. Cabozantinib in patients with advanced and progressing hepatocellular carcinoma. N Engl J Med. 2018; 379:54–63. 10.1056/NEJMoa1717002 29972759 PMC7523244

[9] Zhai B, Sun XY. Mechanisms of resistance to sorafenib and the corresponding strategies in hepatocellular carcinoma. World J Hepatol. 2013; 5:345–52. 10.4254/wjh.v5.i7.345 23898367 PMC3724962

[10] Chen J, Jin R, Zhao J, Liu J, Ying H, Yan H, Zhou S, Liang Y, Huang D, Liang X, Yu H, Lin H, Cai X. Potential molecular, cellular and microenvironmental mechanism of sorafenib resistance in hepatocellular carcinoma. Cancer Lett. 2015; 367:1–11. 10.1016/j.canlet.2015.06.019 26170167

[11] Méndez-Blanco C, Fondevila F, García-Palomo A, González-Gallego J, Mauriz JL. Sorafenib resistance in hepatocarcinoma: role of hypoxia-inducible factors. Exp Mol Med. 2018; 50:1–9. 10.1038/s12276-018-0159-1 30315182 PMC6185986

[12] Leung HW, Lau EYT, Leung CON, Lei MML, Mok EHK, Ma VWS, Cho WCS, Ng IOL, Yun JP, Cai SH, Yu HJ, Ma S, Lee TKW. NRF2/SHH signaling cascade promotes tumor-initiating cell lineage and drug resistance in hepatocellular carcinoma. Cancer Lett. 2020; 476:48–56. 10.1016/j.canlet.2020.02.008 32061952

[13] Kuczynski EA, Vermeulen PB, Pezzella F, Kerbel RS, Reynolds AR. Vessel co-option in cancer. Nat Rev Clin Oncol. 2019; 16:469–93. 10.1038/s41571-019-0181-9 30816337

[14] Kuczynski EA, Yin M, Bar-Zion A, Lee CR, Butz H, Man S, Daley F, Vermeulen PB, Yousef GM, Foster FS, Reynolds AR, Kerbel RS. Co-option of Liver Vessels and Not Sprouting Angiogenesis Drives Acquired Sorafenib Resistance in Hepatocellular Carcinoma. J Natl Cancer Inst. 2016; 108:djw030. 10.1093/jnci/djw030 27059374 PMC5017954

[15] Faivre S, Rimassa L, Finn RS. Molecular therapies for HCC: looking outside the box. J Hepatol. 2020; 72:342–52. 10.1016/j.jhep.2019.09.010 31954496

[16] Wilhelm SM, Dumas J, Adnane L, Lynch M, Carter CA, Schütz G, Thierauch KH, Zopf D. Regorafenib (BAY 73-4506): a new oral multikinase inhibitor of angiogenic, stromal and oncogenic receptor tyrosine kinases with potent preclinical antitumor activity. Int J Cancer. 2011; 129:245–55. 10.1002/ijc.25864 21170960

[17] Liu S, Du Y, Ma H, Liang Q, Zhu X, Tian J. Preclinical comparison of regorafenib and sorafenib efficacy for hepatocellular carcinoma using multimodality molecular imaging. Cancer Lett. 2019; 453:74–83. 10.1016/j.canlet.2019.03.037 30928380

[18] Kissel M, Berndt S, Fiebig L, Kling S, Ji Q, Gu Q, Lang T, Hafner FT, Teufel M, Zopf D. Antitumor effects of regorafenib and sorafenib in preclinical models of hepatocellular carcinoma. Oncotarget. 2017; 8:107096–108. 10.18632/oncotarget.22334 29291014 PMC5739799

[19] Zhou L, Huang Y, Li J, Wang Z. The mTOR pathway is associated with the poor prognosis of human hepatocellular carcinoma. Med Oncol. 2010; 27:255–61. 10.1007/s12032-009-9201-4 19301157

[20] Dong J, Zhai B, Sun W, Hu F, Cheng H, Xu J. Activation of phosphatidylinositol 3-kinase/AKT/snail signaling pathway contributes to epithelial-mesenchymal transition-induced multi-drug resistance to sorafenib in hepatocellular carcinoma cells. PLoS One. 2017; 12:e0185088. 10.1371/journal.pone.0185088 28934275 PMC5608310

[21] Zhai B, Hu F, Jiang X, Xu J, Zhao D, Liu B, Pan S, Dong X, Tan G, Wei Z, Qiao H, Jiang H, Sun X. Inhibition of Akt reverses the acquired resistance to sorafenib by switching protective autophagy to autophagic cell death in hepatocellular carcinoma. Mol Cancer Ther. 2014; 13:1589–98. 10.1158/1535-7163.MCT-13-1043 24705351

[22] Zhai B, Hu F, Yan H, Zhao D, Jin X, Fang T, Pan S, Sun X, Xu L. Bufalin reverses resistance to sorafenib by inhibiting Akt activation in hepatocellular carcinoma: the role of endoplasmic reticulum stress. PLoS One. 2015; 10:e0138485. 10.1371/journal.pone.0138485 26381511 PMC4575108

[23] Zhai B, Jiang X, He C, Zhao D, Ma L, Xu L, Jiang H, Sun X. Arsenic trioxide potentiates the anti-cancer activities of sorafenib against hepatocellular carcinoma by inhibiting Akt activation. Tumour Biol. 2015; 36:2323–34. 10.1007/s13277-014-2839-3 25416439

[24] Leung CO, Tong M, Chung KP, Zhou L, Che N, Tang KH, Ding J, Lau EY, Ng IO, Ma S, Lee TK. Overriding adaptive resistance to sorafenib through combination therapy with src homology 2 domain-containing phosphatase 2 blockade in hepatocellular carcinoma. Hepatology. 2020; 72:155–68. 10.1002/hep.30989 31610028

[25] Toh TB, Lim JJ, Hooi L, Rashid MB, Chow EK. Targeting Jak/Stat pathway as a therapeutic strategy against SP/CD44+ tumorigenic cells in Akt/β-catenin-driven hepatocellular carcinoma. J Hepatol. 2020; 72:104–18. 10.1016/j.jhep.2019.08.035 31541681

[26] Dietrich P, Koch A, Fritz V, Hartmann A, Bosserhoff AK, Hellerbrand C. Wild type Kirsten rat sarcoma is a novel microRNA-622-regulated therapeutic target for hepatocellular carcinoma and contributes to sorafenib resistance. Gut. 2018; 67:1328–41. 10.1136/gutjnl-2017-315402 29275358

[27] Masuda M, Chen WY, Miyanaga A, Nakamura Y, Kawasaki K, Sakuma T, Ono M, Chen CL, Honda K, Yamada T. Alternative mammalian target of rapamycin (mTOR) signal activation in sorafenib-resistant hepatocellular carcinoma cells revealed by array-based pathway profiling. Mol Cell Proteomics. 2014; 13:1429–38. 10.1074/mcp.M113.033845 24643969 PMC4047464

[28] Rebouissou S, Nault JC. Advances in molecular classification and precision oncology in hepatocellular carcinoma. J Hepatol. 2020; 72:215–29. 10.1016/j.jhep.2019.08.017 31954487

[29] Llovet JM, Hernandez-Gea V. Hepatocellular carcinoma: reasons for phase III failure and novel perspectives on trial design. Clin Cancer Res. 2014; 20:2072–79. 10.1158/1078-0432.CCR-13-0547 24589894

[30] Yap TA, Yan L, Patnaik A, Fearen I, Olmos D, Papadopoulos K, Baird RD, Delgado L, Taylor A, Lupinacci L, Riisnaes R, Pope LL, Heaton SP, et al. First-in-man clinical trial of the oral pan-AKT inhibitor MK-2206 in patients with advanced solid tumors. J Clin Oncol. 2011; 29:4688–95. 10.1200/JCO.2011.35.5263 22025163

[31] Matter MS, Decaens T, Andersen JB, Thorgeirsson SS. Targeting the mTOR pathway in hepatocellular carcinoma: current state and future trends. J Hepatol. 2014; 60:855–65. 10.1016/j.jhep.2013.11.031 24308993 PMC3960348

[32] Ahmed SM, Thériault BL, Uppalapati M, Chiu CW, Gallie BL, Sidhu SS, Angers S. KIF14 negatively regulates Rap1a-radil signaling during breast cancer progression. J Cell Biol. 2012; 199:951–67. 10.1083/jcb.201206051 23209302 PMC3518219

[33] Yang Z, Li C, Yan C, Li J, Yan M, Liu B, Zhu Z, Wu Y, Gu Q. KIF14 promotes tumor progression and metastasis and is an independent predictor of poor prognosis in human gastric cancer. Biochim Biophys Acta Mol Basis Dis. 2019; 1865:181–92. 10.1016/j.bbadis.2018.10.039 30404039

[34] Wang ZZ, Yang J, Jiang BH, Di JB, Gao P, Peng L, Su XQ. KIF14 promotes cell proliferation via activation of Akt and is directly targeted by miR-200c in colorectal cancer. Int J Oncol. 2018; 53:1939–52. 10.3892/ijo.2018.4546 30226594 PMC6192758

[35] Xu H, Choe C, Shin SH, Park SW, Kim HS, Jung SH, Yim SH, Kim TM, Chung YJ. Silencing of KIF14 interferes with cell cycle progression and cytokinesis by blocking the p27(Kip1) ubiquitination pathway in hepatocellular carcinoma. Exp Mol Med. 2014; 46:e97. 10.1038/emm.2014.23 24854087 PMC4044675

[36] Yang T, Zhang XB, Zheng ZM. Suppression of KIF14 expression inhibits hepatocellular carcinoma progression and predicts favorable outcome. Cancer Sci. 2013; 104:552–57. 10.1111/cas.12128 23414349 PMC7657169

[37] Huang W, Wang J, Zhang D, Chen W, Hou L, Wu X, Lu Y. Inhibition of KIF14 suppresses tumor cell growth and promotes apoptosis in human glioblastoma. Cell Physiol Biochem. 2015; 37:1659–70. 10.1159/000438532 26536004

[38] Singel SM, Cornelius C, Zaganjor E, Batten K, Sarode VR, Buckley DL, Peng Y, John GB, Li HC, Sadeghi N, Wright WE, Lum L, Corson TW, Shay JW. KIF14 promotes AKT phosphorylation and contributes to chemoresistance in triple-negative breast cancer. Neoplasia. 2014; 16:247–56, 256.e2. 10.1016/j.neo.2014.03.008 24784001 PMC4094827

[39] Singel SM, Cornelius C, Batten K, Fasciani G, Wright WE, Lum L, Shay JW. A targeted RNAi screen of the breast cancer genome identifies KIF14 and TLN1 as genes that modulate docetaxel chemosensitivity in triple-negative breast cancer. Clin Cancer Res. 2013; 19:2061–70. 10.1158/1078-0432.CCR-13-0082 23479679 PMC4513911

[40] Ito Y, Miyoshi E, Takeda T, Sakon M, Noda K, Tsujimoto M, Monden M, Taniguchi N, Matsuura N. Expression and possible role of ets-1 in hepatocellular carcinoma. Am J Clin Pathol. 2000; 114:719–25. 10.1309/RAVV-8NM1-CJB7-GJFR 11068545

[41] Ozaki I, Mizuta T, Zhao G, Yotsumoto H, Hara T, Kajihara S, Hisatomi A, Sakai T, Yamamoto K. Involvement of the Ets-1 gene in overexpression of matrilysin in human hepatocellular carcinoma. Cancer Res. 2000; 60:6519–25. 11103822

[42] Kidger AM, Keyse SM. The regulation of oncogenic Ras/ERK signalling by dual-specificity mitogen activated protein kinase phosphatases (MKPs). Semin Cell Dev Biol. 2016; 50:125–32. 10.1016/j.semcdb.2016.01.009 26791049 PMC5056954

[43] Phuchareon J, McCormick F, Eisele DW, Tetsu O. EGFR inhibition evokes innate drug resistance in lung cancer cells by preventing Akt activity and thus inactivating Ets-1 function. Proc Natl Acad Sci USA. 2015; 112:E3855–63. 10.1073/pnas.1510733112 26150526 PMC4517222

[44] Xu H, Zhao G, Zhang Y, Jiang H, Wang W, Zhao D, Yu H, Qi L. Long non-coding RNA PAXIP1-AS1 facilitates cell invasion and angiogenesis of glioma by recruiting transcription factor ETS1 to upregulate KIF14 expression. J Exp Clin Cancer Res. 2019; 38:486. 10.1186/s13046-019-1474-7 31823805 PMC6902534

[45] Shao Z, Li Y, Dai W, Jia H, Zhang Y, Jiang Q, Chai Y, Li X, Sun H, Yang R, Cao Y, Feng F, Guo Y. ETS-1 induces Sorafenib-resistance in hepatocellular carcinoma cells via regulating transcription factor activity of PXR. Pharmacol Res. 2018; 135:188–200. 10.1016/j.phrs.2018.08.003 30114438

[46] Han P, Li H, Jiang X, Zhai B, Tan G, Zhao D, Qiao H, Liu B, Jiang H, Sun X. Dual inhibition of Akt and c-Met as a second-line therapy following acquired resistance to sorafenib in hepatocellular carcinoma cells. Mol Oncol. 2017; 11:320–34. 10.1002/1878-0261.12039 28164434 PMC5527443

[47] Cao SS, Zhen YS. Potentiation of antimetabolite antitumor activity *in vivo* by dipyridamole and amphotericin B. Cancer Chemother Pharmacol. 1989; 24:181–86. 10.1007/BF00300240 2736709

[48] Cohen JD, Tham KY, Mastrandrea NJ, Gallegos AC, Monks TJ, Lau SS. cAMP-dependent cytosolic mislocalization of p27(kip)-cyclin D1 during quinol-thioether-induced tuberous sclerosis renal cell carcinoma. Toxicol Sci. 2011; 122:361–71. 10.1093/toxsci/kfr118 21693435 PMC3155088

[49] Hage C, Hoves S, Strauss L, Bissinger S, Prinz Y, Pöschinger T, Kiessling F, Ries CH. Sorafenib induces pyroptosis in macrophages and triggers natural killer cell-mediated cytotoxicity against hepatocellular carcinoma. Hepatology. 2019; 70:1280–97. 10.1002/hep.30666 31002440

[50] Bhagyaraj E, Ahuja N, Kumar S, Tiwari D, Gupta S, Nanduri R, Gupta P. TGF-β induced chemoresistance in liver cancer is modulated by xenobiotic nuclear receptor PXR. Cell Cycle. 2019; 18:3589–602. 10.1080/15384101.2019.1693120 31739702 PMC6927732

[51] Roof AK, Gutierrez-Hartmann A. Consider the context: Ras/ERK and PI3K/AKT/mTOR signaling outcomes are pituitary cell type-specific. Mol Cell Endocrinol. 2018; 463:87–96. 10.1016/j.mce.2017.04.019 28445712

[52] Wu M, Liu X, Jin W, Li Y, Li Y, Hu Q, Chu PK, Tang G, Ping Y. Targeting ETS1 with RNAi-based supramolecular nanoassemblies for multidrug-resistant breast cancer therapy. J Control Release. 2017; 253:110–21. 10.1016/j.jconrel.2017.03.011 28302581

[53] Liu T, Liu R, Zhang S, Guo K, Zhang Q, Li W, Liu Y. Sorafenib induced alteration of protein glycosylation in hepatocellular carcinoma cells. Oncol Lett. 2017; 14:517–24. 10.3892/ol.2017.6177 28693200 PMC5494657

[54] Zhao D, Zhai B, He C, Tan G, Jiang X, Pan S, Dong X, Wei Z, Ma L, Qiao H, Jiang H, Sun X. Upregulation of HIF-2α induced by sorafenib contributes to the resistance by activating the TGF-α/EGFR pathway in hepatocellular carcinoma cells. Cell Signal. 2014; 26:1030–39. 10.1016/j.cellsig.2014.01.026 24486412

[55] Chen W, Yang J, Zhang Y, Cai H, Chen X, Sun D. Regorafenib reverses HGF-induced sorafenib resistance by inhibiting epithelial-mesenchymal transition in hepatocellular carcinoma. FEBS Open Bio. 2019; 9:335–47. 10.1002/2211-5463.12578 30761258 PMC6356182

[56] Wilhelm SM, Carter C, Tang L, Wilkie D, McNabola A, Rong H, Chen C, Zhang X, Vincent P, McHugh M, Cao Y, Shujath J, Gawlak S, et al. BAY 43-9006 exhibits broad spectrum oral antitumor activity and targets the RAF/MEK/ERK pathway and receptor tyrosine kinases involved in tumor progression and angiogenesis. Cancer Res. 2004; 64:7099–109. 10.1158/0008-5472.CAN-04-1443 15466206

[57] Sishtla K, Pitt N, Shadmand M, O’Hare MN, Sulaiman RS, Sinn AL, Condon K, Pollok KE, Sandusky GE, Corson TW. Observations on spontaneous tumor formation in mice overexpressing mitotic kinesin Kif14. Sci Rep. 2018; 8:16152. 10.1038/s41598-018-34603-4 30385851 PMC6212535

[58] Mendoza MC, Er EE, Blenis J. The Ras-ERK and PI3K-mTOR pathways: cross-talk and compensation. Trends Biochem Sci. 2011; 36:320–28. 10.1016/j.tibs.2011.03.006 21531565 PMC3112285

[59] Dittmer J. The role of the transcription factor Ets1 in carcinoma. Semin Cancer Biol. 2015; 35:20–38. 10.1016/j.semcancer.2015.09.010 26392377

[60] Lavenburg KR, Ivey J, Hsu T, Muise-Helmericks RC. Coordinated functions of Akt/PKB and ETS1 in tubule formation. FASEB J. 2003; 17:2278–80. 10.1096/fj.03-0040fje 14525946 PMC2276577

[61] Pang D, Wang L, Dong J, Lai X, Huang Q, Milner R, Li L. Integrin α5β1-Ang1/Tie2 receptor cross-talk regulates brain endothelial cell responses following cerebral ischemia. Exp Mol Med. 2018; 50:117. 10.1038/s12276-018-0145-7 30185785 PMC6123805

[62] Sato Y. Role of ETS family transcription factors in vascular development and angiogenesis. Cell Struct Funct. 2001; 26:19–24. 10.1247/csf.26.19 11345500

[63] Tsao HW, Tai TS, Tseng W, Chang HH, Grenningloh R, Miaw SC, Ho IC. Ets-1 facilitates nuclear entry of NFAT proteins and their recruitment to the IL-2 promoter. Proc Natl Acad Sci USA. 2013; 110:15776–81. 10.1073/pnas.1304343110 24019486 PMC3785780

[64] Liu F, Cao J, Wu J, Sullivan K, Shen J, Ryu B, Xu Z, Wei W, Cui R. Stat3-targeted therapies overcome the acquired resistance to vemurafenib in melanomas. J Invest Dermatol. 2013; 133:2041–49. 10.1038/jid.2013.32 23344460 PMC9744462

[65] Potu H, Peterson LF, Kandarpa M, Pal A, Sun H, Durham A, Harms PW, Hollenhorst PC, Eskiocak U, Talpaz M, Donato NJ. Usp9x regulates Ets-1 ubiquitination and stability to control NRAS expression and tumorigenicity in melanoma. Nat Commun. 2017; 8:14449. 10.1038/ncomms14449 28198367 PMC5316860

[66] Clough E, Barrett T. The gene expression omnibus database. Methods Mol Biol. 2016; 1418:93–110. 10.1007/978-1-4939-3578-9_5 27008011 PMC4944384

[67] Law V, Knox C, Djoumbou Y, Jewison T, Guo AC, Liu Y, Maciejewski A, Arndt D, Wilson M, Neveu V, Tang A, Gabriel G, Ly C, et al. DrugBank 4.0: shedding new light on drug metabolism. Nucleic Acids Res. 2014; 42:D1091–97. 10.1093/nar/gkt1068 24203711 PMC3965102

[68] Weinstein JN, Collisson EA, Mills GB, Shaw KR, Ozenberger BA, Ellrott K, Shmulevich I, Sander C, Stuart JM, and Cancer Genome Atlas Research Network. The cancer genome atlas pan-cancer analysis project. Nat Genet. 2013; 45:1113–20. 10.1038/ng.2764 24071849 PMC3919969

[69] Dennis G Jr, Sherman BT, Hosack DA, Yang J, Gao W, Lane HC, Lempicki RA. DAVID: database for annotation, visualization, and integrated discovery. Genome Biol. 2003; 4:P3. 12734009

[70] Szklarczyk D, Franceschini A, Wyder S, Forslund K, Heller D, Huerta-Cepas J, Simonovic M, Roth A, Santos A, Tsafou KP, Kuhn M, Bork P, Jensen LJ, von Mering C. STRING v10: protein-protein interaction networks, integrated over the tree of life. Nucleic Acids Res. 2015; 43:D447–52. 10.1093/nar/gku1003 25352553 PMC4383874

[71] Kohl M, Wiese S, Warscheid B. Cytoscape: software for visualization and analysis of biological networks. Methods Mol Biol. 2011; 696:291–303. 10.1007/978-1-60761-987-1_18 21063955

[72] Ma L, Zhai B, Zhu H, Li W, Jiang W, Lei L, Zhang S, Qiao H, Jiang X, Sun X. The miR-141/neuropilin-1 axis is associated with the clinicopathology and contributes to the growth and metastasis of pancreatic cancer. Cancer Cell Int. 2019; 19:248. 10.1186/s12935-019-0963-2 31572065 PMC6764122

