# Supplementary material for: Silencing KIF14 reverses acquired resistance to sorafenib in hepatocellular carcinoma
Source: Aging (Albany NY). 2020 Nov 16;12(22):22975–3003. doi: 10.18632/aging.104028 (PMC7746348; doi:10.18632/aging.104028)
Supplement: Supplementary Figures [file aging-12-104028-s001..pdf]

SUPPLEMENTARY FIGURES

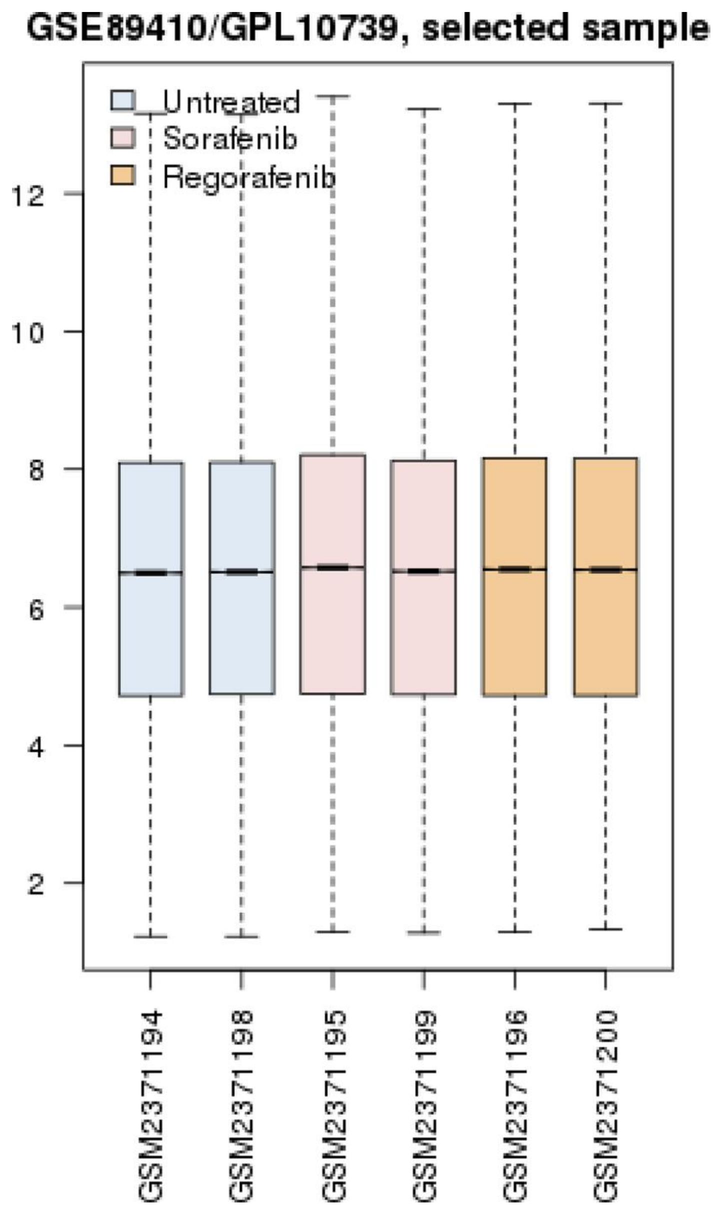

Supplementary Figure 1. DEGs from the GEO database. The gene expression profiles of GSE89410 were downloaded from the GEO database (http://www.ncbi.nlm.nih.gov/geo/), which consists of data from patient-derived pluripotent stem cells treated with 1 µM sorafenib, 1 µM regorafenib and no treatment controls. DEGs, differentially expressed genes; GEO, Gene Expression Omnibus.

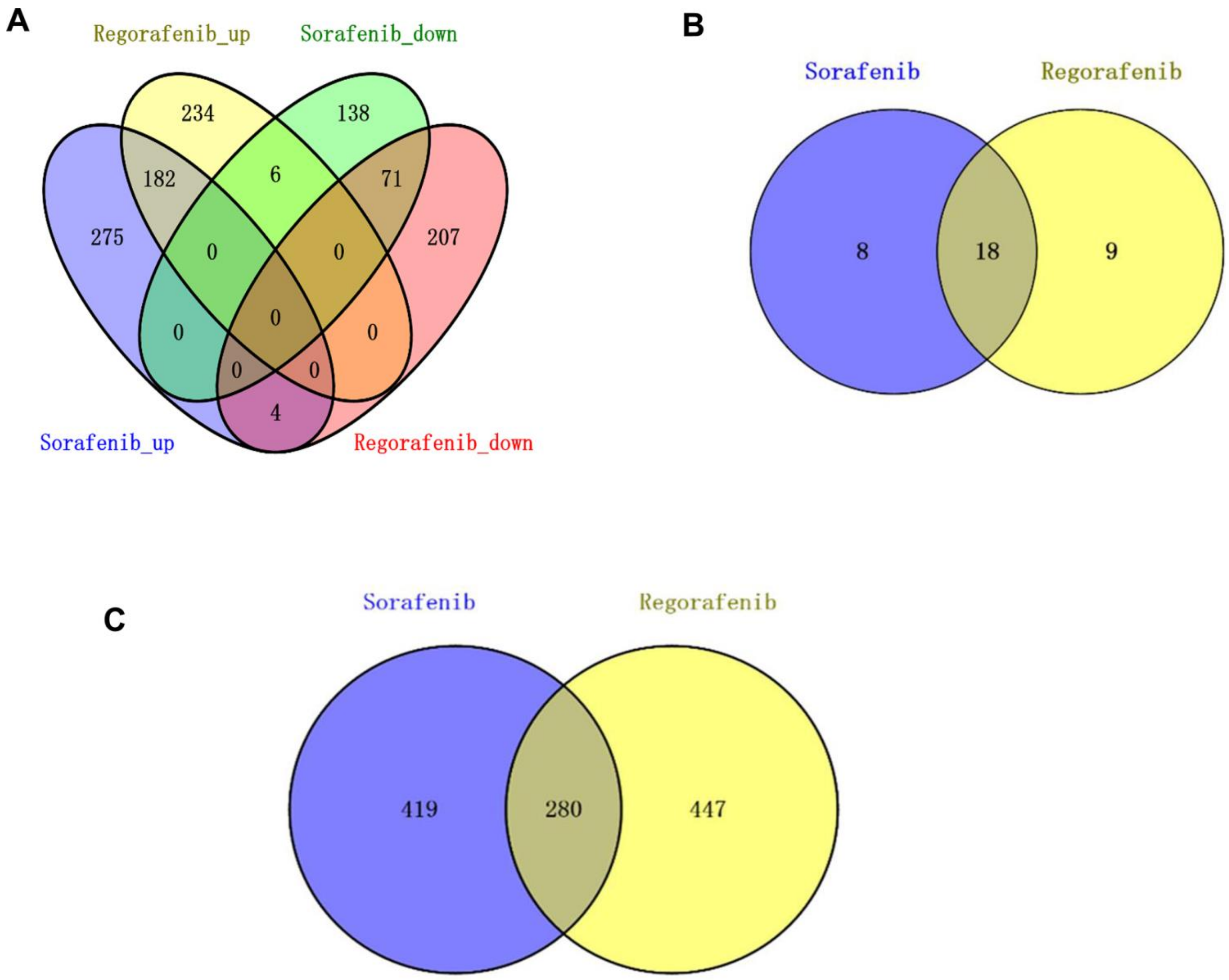

Supplementary Figure 2. DEGs identified from the GEO and DrugBank databases. (**A**) DEGs downloaded from the GSE89410 dataset. (**B**) Genes induced by sorafenib and regorafenib obtained from the DrugBank database (https://www.drugbank.ca/). (**C**) Genes induced by sorafenib and regorafenib from (**A**) and (**B**) integrated into a Venn diagram (online tool: http://bioinformatics.psb.ugent.be/webtools/Venn/). DEGs, differentially expressed genes; GEO, Gene Expression Omnibus.

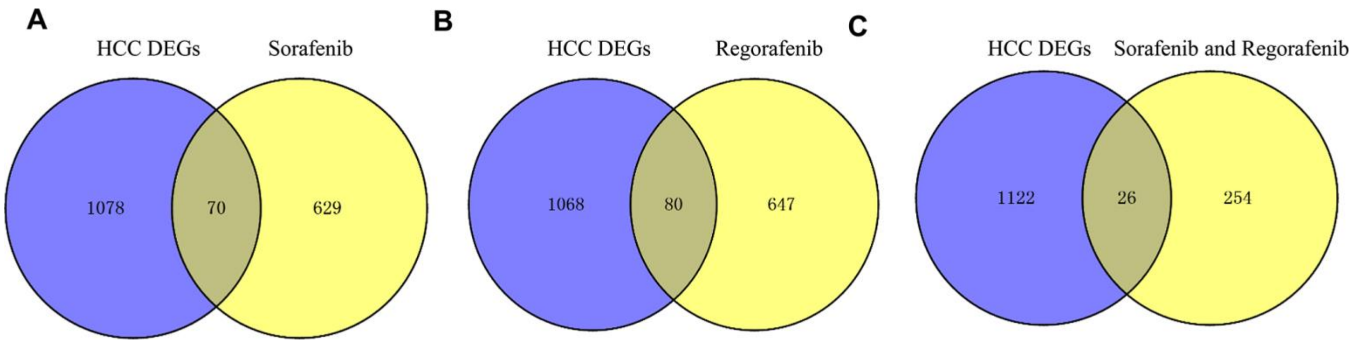

Supplementary Figure 3. DEGs induced by sorafenib and regorafenib in HCC. DEGs in HCC were downloaded from The Cancer Genome Atlas (https://cancergenome.nih.gov/). (**A**) DEGs in HCC and the sorafenib target genes identified in a Venn diagram. (**B**) DEGs in HCC and the regorafenib target genes were identified in a Venn diagram. (**C**) DEGs in HCC and the intersection of the sorafenib and regorafenib target genes identified in a Venn diagram. DEGs, differentially expressed genes; HCC, hepatocellular carcinoma.

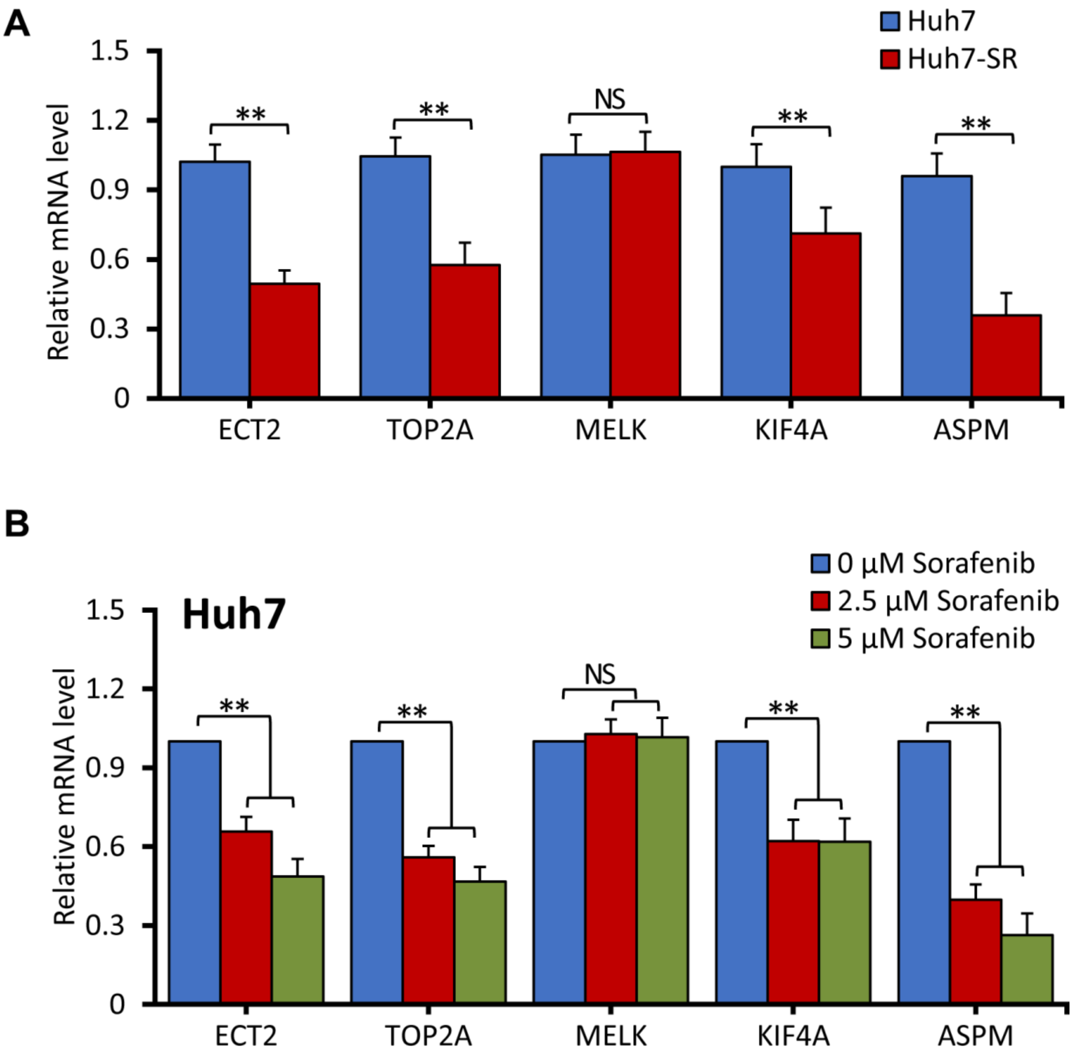

Supplementary Figure 4. DEGs from bioinformatics analysis identified using qRT-PCR. (**A**) Gene expression in sorafenib-resistant Huh7-SR and HepG2-SR cells and the corresponding parental cells detected with qRT-PCR. (**B**) Huh7 cells were incubated for 48 h with 0, 2.5 or 5 μM sorafenib. mRNA expression was measured by qRT-PCR and normalized to GAPDH. The level of mRNA from untreated cells was assigned a value of 1. Data represent three independent experiments. NS, not significant. "**" indicates P<0.001. DEGs, differentially expressed genes; qRT-PCR, quantitative reverse-transcription polymerase chain reaction.

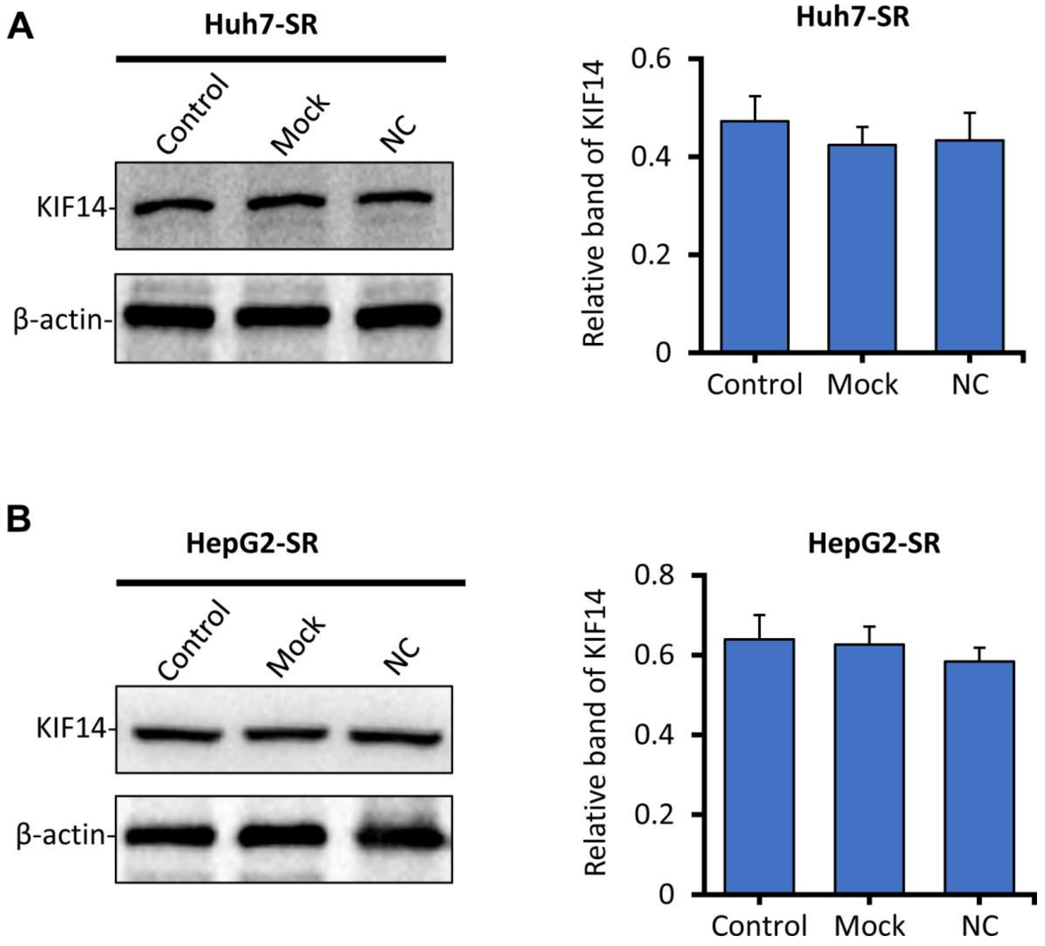

Supplementary Figure 5. KIF14 protein expression profile is unaffected by NC. Huh7-SR (**A**) and HepG2-SR (**B**) cells were transfected for 48 h with NC, after which western blot analysis was used to assess the protein expression profiles. The density of each band was normalized to that of β-actin. The transfection reagents served as a mock control. Corresponding untransfected cells served as a control. Data represent three independent experiments. NC, negative control.

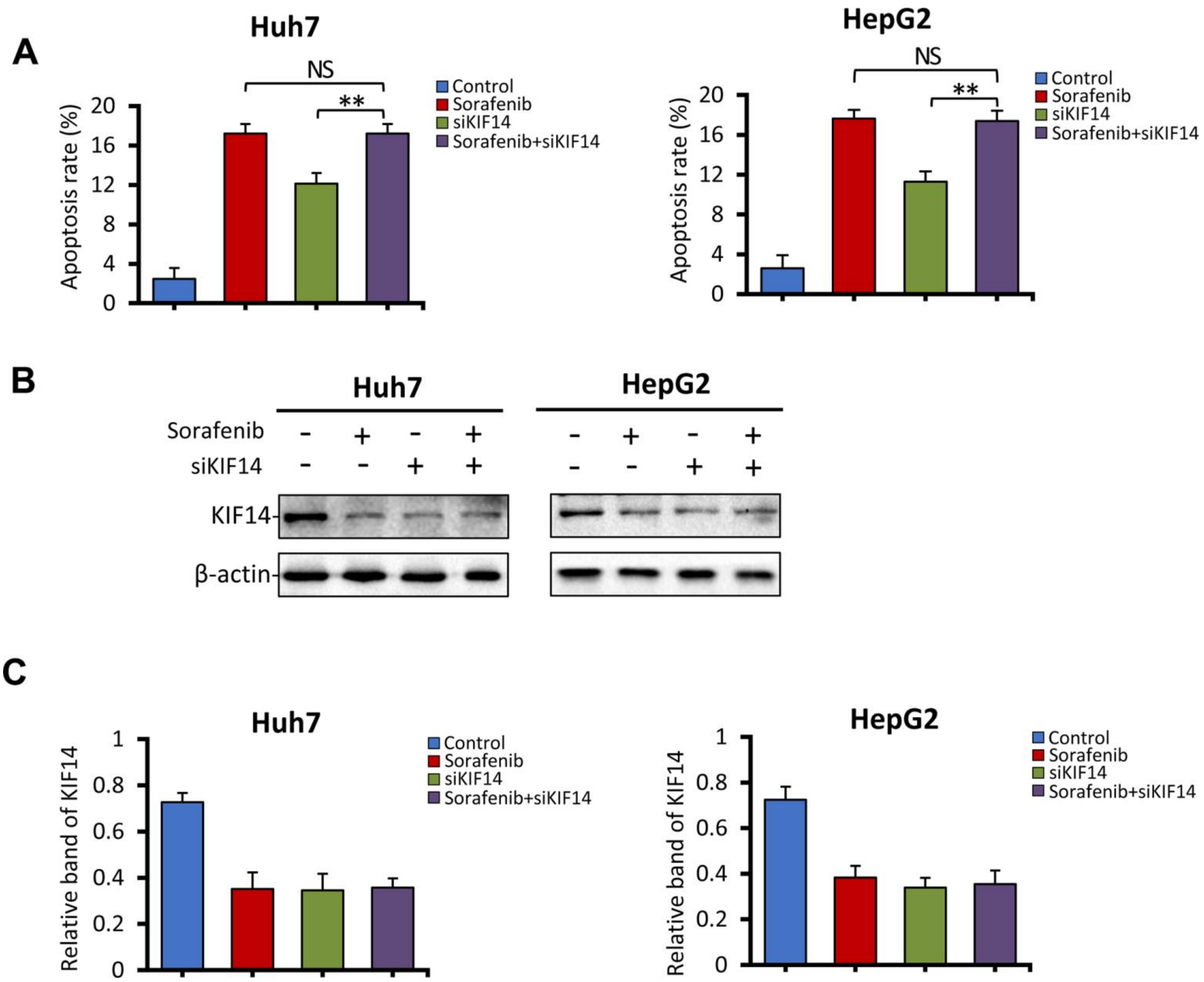

Supplementary Figure 6. Silencing KIF14 does not have a synergistic effect with sorafenib in parental HCC cells. (**A**) Huh7 and HepG2 cells were transfected for 24 h with control or siKIF14 and then incubated for 24 h with 0 or 5 μM sorafenib. Cells were then analyzed cytometrically to detect apoptosis, and the rates of apoptosis were determined. (**B**, **C**) Cells from (**A**) were subjected to western blot analysis to assess protein expression profiles (**B**). The density of each band was normalized to that of β-actin (**C**). Data represent three independent experiments. NS, not significant. "**" indicates P<0.001.
