# Supplementary material for: Silencing KIF14 reverses acquired resistance to sorafenib in hepatocellular carcinoma
Source: Aging (Albany NY). 2020 Nov 16;12(22):22975–3003. doi: 10.18632/aging.104028 (PMC7746348; doi:10.18632/aging.104028)
Supplement: Supplementary Tables [file aging-12-104028-s002..pdf]

SUPPLEMENTARY TABLES

Supplementary Table 1. Top 10 enriched KEGG pathways of sorafenib in HCC.

Category	Term	Count	P Value
KEGG	hsa00830:Retinol metabolism	7	8.55E-07
KEGG	hsa00982:Drug metabolism - cytochrome P450	7	1.12E-06
KEGG	hsa05204:Chemical carcinogenesis	7	2.94E-06
KEGG	hsa00591:Linoleic acid metabolism	5	1.34E-05
KEGG	hsa00980:Metabolism of xenobiotics by cytochrome P450	5	5.51E-04
KEGG	hsa00140:Steroid hormone biosynthesis	4	3.29E-03
KEGG	hsa00590:Arachidonic acid metabolism	4	3.98E-03
KEGG	hsa00983:Drug metabolism - other enzymes	3	2.37E-02
KEGG	hsa00232:Caffeine metabolism	2	2.58E-02
KEGG	hsa01100:Metabolic pathways	12	4.29E-02

Supplementary Table 2. Top 10 enriched KEGG pathways of regorafenib in HCC.

Category	Term	Count	P Value
KEGG	hsa04151:PI3K-Akt signaling pathway	15	4.72E-08
KEGG	hsa00982:Drug metabolism - cytochrome P450	8	3.49E-07
KEGG	hsa00830:Retinol metabolism	7	4.93E-06
KEGG	hsa05204:Chemical carcinogenesis	6	2.13E-04
KEGG	hsa04512:ECM-receptor interaction	6	3.15E-04
KEGG	hsa00591:Linoleic acid metabolism	4	1.01E-03
KEGG	hsa00980:Metabolism of xenobiotics by cytochrome P450	5	1.65E-03
KEGG	hsa04510:Focal adhesion	7	2.82E-03
KEGG	hsa04015:Rap1 signaling pathway	7	3.10E-03
KEGG	hsa00590:Arachidonic acid metabolism	4	8.93E-03

Supplementary Table 3. The genes related to the survival of HCC patients in PPI network.

Gene	Logrank p value	Gene	Logrank p value	Gene	Logrank p value	Gene	Logrank p value
**KIF2C**	0.000011	MELK	0.0015	KIF14	0.0052	SHCBP1	0.018
**EXO1**	0.00034	DNA2	0.0015	TEK	0.0052	CYP3A4	0.019
**ASPM**	0.00061	PDE2A	0.0017	CYP2C8	0.0064	CDC6	0.02
**CYP2C9**	0.0007	PLK4	0.0028	KNTC1	0.0072	NCAPH	0.02
**ECT2**	0.00076	BUB1B	0.0028	CCNE2	0.0072	ITGA2	0.022
**ANLN**	0.00085	TOP2A	0.0028	ABCA9	0.0075	ESPL1	0.024
**TYMS**	0.0009	POLQ	0.0033	TCF19	0.01	ZNF692	0.024
**KIF4A**	0.001	MYBL2	0.0034	TARBP1	0.013	COL15A1	0.037
**KIF15**	0.0011	TROAP	0.0035	MSH5	0.014		
**STMN1**	0.0011	DTL	0.0049	XDH	0.015		

Abbreviations: PPI, protein-protein interaction.

Supplementary Table 4. Top10 highest degree proteins of sorafenib and regorafenib in PPI network.

SORAFENIB			REGORAFENIB		
**Node**	**Type**	**Degree**	**Node**	**Type**	**Degree**
**EXO1**	down	14	**TOP2A**	down	22
**TYMS**	down	14	**CDKN3**	down	16
**POLQ**	down	13	**MELK**	down	16
**PLK4**	down	13	**KIF2C**	down	15
**UBE2C**	down	12	**ASPM**	down	15
**BUB1B**	down	12	**KIF4A**	down	14
**DTL**	down	12	**KIF14**	down	14
**KIF15**	down	12	**LRRK2**	up	14
**ANLN**	down	12	**TYMS**	down	13
**CDC6**	down	11	**KNTC1**	down	13

Supplementary Table 5. The CDIs of siKIF14 in combination with sorafenib in Huh7-SR cells.

Sorafenib (μM)	siKIF4-2	siKIF4-3
2.5	0.99	0.98
5	0.69	0.69
10	0.72	0.75

Abbreviations: CDI, coefficient of drug interaction.

Supplementary Table 6. The CDIs of siKIF14 in combination with sorafenib in HepG2-SR cells.

Sorafenib (μM)	siKIF4-2	siKIF4-3
2.5	0.97	0.95
5	0.67	0.66
10	0.71	0.73

Abbreviations: CDI, coefficient of drug interaction.

Supplementary Table 7. The primers used for RT-PCR in this study.

Genes	Gene ID	Primers (5' to 3')
ECT2	1894	Forward: GCAAGAGTGGTTCTGGGGAA<br>Reverse: TTGCGATTGCTGTTAGGGGT
TOP2A	7153	Forward: GAAGTGTCACCATTGCAGCC<br>Reverse: TGTCTGGGCGGAGCAAAATA
MELK	9833	Forward: AGATGTTTGGAGCATGGGCA<br>Reverse: ATGCTACTGGGAGAGAGCCA
KIF4A	24137	Forward: ACGCCATCTGAATGACCTCC<br>Reverse: CCACGCACTTCAGTAAGGGA
ASPM	259266	Forward: ATCATCCTGCAATCTAGGATAAGAA<br>Reverse: AAATAAGCACGCCAATGCCTC
